# The Matrix Protein Tropoelastin Prolongs Mesenchymal Stromal Cell Vitality and Delays Senescence During Replicative Aging

**DOI:** 10.1002/advs.202402168

**Published:** 2024-08-09

**Authors:** Sunny Shinchen Lee, Aleen Al Halawani, Jonathan D. Teo, Anthony S. Weiss, Giselle C. Yeo

**Affiliations:** ^1^ School of Life & Environmental Sciences and Charles Perkins Centre The University of Sydney Camperdown NSW 2006 Australia; ^2^ School of Medical Sciences and Charles Perkins Centre The University of Sydney Camperdown NSW 2006 Australia; ^3^ Sydney Nano Institute The University of Sydney Camperdown NSW 2006 Australia

**Keywords:** aging, fitness, mesenchymal stromal cells, senescence, stem cells, tropoelastin

## Abstract

Cellular senescence leads to the functional decline of regenerative cells such as mesenchymal stromal/stem cells (MSCs), which gives rise to chronic conditions and contributes to poor cell therapy outcomes. Aging tissues are associated with extracellular matrix (ECM) dysregulation, including loss of elastin. However, the role of the ECM in modulating senescence is underexplored. In this work, it is shown that tropoelastin, the soluble elastin precursor, is not only a marker of young MSCs but also actively preserves cell fitness and delays senescence during replicative aging. MSCs briefly exposed to tropoelastin exhibit upregulation of proliferative genes and concurrent downregulation of senescence genes. The seno‐protective benefits of tropoelastin persist during continuous, long‐term MSC culture, and significantly extend the MSC replicative lifespan. Tropoelastin‐expanded MSCs further maintain youth‐associated phenotype and function compared to age‐matched controls, including preserved clonogenic potential, minimal senescence‐associated beta‐galactosidase activity, maintained cell sizes, reduced expression of senescence markers, suppressed secretion of senescence‐associated factors, and increased production of youth‐associated proteins. This work points to the utility of exogenously‐supplemented tropoelastin for manufacturing MSCs that robustly maintain regenerative potential with age. It further reveals the active role of classical structural ECM proteins in driving cellular age‐associated fitness, potentially leading to future interventions for aging‐related pathologies.

## Graphical abstract

Consistent with elastin loss during organismal aging, the extracellular matrix component tropoelastin is downregulated during cellular replicative senescence. Exogenous supplementation of tropoelastin significantly reduces senescent phenotypes and prolongs functional vigor in aging mesenchymal stromal cells. This study reveals that the extracellular matrix drives fitness during cellular aging, and serves as a potential target in regenerative medicine approaches for aging‐related pathologies.

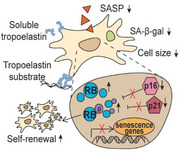

## 1 Introduction

Senescence is classically defined as a state of permanent cell cycle arrest in metabolically active cells, in response to cell‐damaging stimuli.^[^ ^1^ ^]^ Controlled senescence plays an important role throughout the lifespan of an organism through effects in embryogenesis, morphogenesis, and tissue regeneration.^[^ ^2^ ^]^ However, the accumulation of senescent cells also forms the basis of tissue degeneration and aging.^[^ ^1^ ^]^ It is thought that senescent stem cells or progenitor cells contribute to degenerative conditions such as osteoarthritis,^[^ ^3^ ^]^ multiple sclerosis,^[^ ^4^ ^]^ and sarcopenia.^[^ ^5^ ^]^ Stem cell populations, including mesenchymal stromal/stem cells (MSCs), are highly susceptible to undergoing senescence when requisitely expanded in vitro for downstream applications. Senescing MSCs exhibit a decline in regenerative capabilities, including self‐renewal, differentiation, and paracrine secretion, which are thought to underpin the therapeutic actions of these cells.^[^ ^6^, ^7^ ^]^ Consequently, cellular senescence poses a major barrier to achieving consistent efficacy in MSC therapies.^[^ ^8^, ^9^ ^]^

MSCs are highly sensitive to their immediate environment.^[^ ^10^ ^]^ Current efforts to delay or counteract MSC senescence have focused on emulating the biological niche via alterations to oxygen tension,^[^ ^11^, ^12^ ^]^ substrate stiffness,^[^ ^13^ ^]^ or concentrations of soluble factors.^[^ ^14^ ^]^ However, despite links between dysregulated extracellular matrix (ECM) components and cellular senescence in disease and aging,^[^ ^15^, ^16^ ^]^ the biological role and application of ECM proteins in preserving MSC age‐related fitness are underexplored.

Tropoelastin is the soluble monomer of elastin, which assembles into elastic fibers in the ECM to confer strength and resilience to elastic tissues. Tropoelastin influences the development of tissues derived from the mesoderm,^[^ ^17^ ^]^ and is traditionally ascribed a structural role.^[^ ^18^, ^19^ ^]^ Over the last few years, tropoelastin has been demonstrated to exert cell‐instructive capabilities, including mitogenic effects on hematopoietic stem cells and MSCs that parallel the potency of growth factors.^[^ ^20^, ^21^ ^]^ While strong proliferative cues are presumed to accelerate replicative stress and promote cellular senescence,^[^ ^22^ ^]^ biologically, there is evidence that tropoelastin may act to the contrary. Peak tropoelastin expression occurs during early development,^[^ ^23^, ^24^ ^]^ and loss of elastin is a hallmark of aging skin and vasculature.^[^ ^25^ ^]^

This work aims to investigate the effects of tropoelastin on MSC senescence. We demonstrate that tropoelastin supplemented as a culture substrate or a soluble media additive can effectively delay various hallmarks of MSC senescence in vitro, including the age‐related decline in clonogenic potential, increase in senescence‐associated beta‐galactosidase (SA *β‐*gal) activity, upregulation of senescence genes, and acquisition of senescence‐associated secretory phenotype (SASP). Our work suggests that matrix proteins can be potent modulators of cellular senescence and alter the progression of senescence phenotypes and dysfunction. These seno‐protective effects of tropoelastin point to its potential use, alongside other cell niche components, to preserve the regenerative function of MSCs, and to improve the feasibility and efficacy of stem cell applications.

## 2 Results

### 2.1 Tropoelastin Simultaneously Promotes Cell Cycle Pathways and Downregulates Senescence Genes

**Figure 1:**
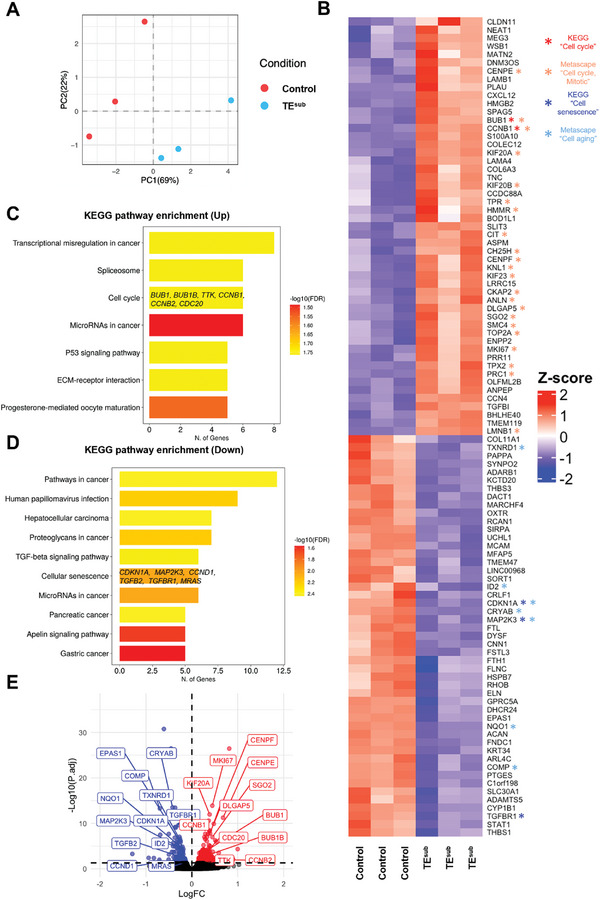
Bulk RNA sequencing reveals tropoelastin‐mediated upregulation of cell cycle genes and downregulation of genes associated with senescence. A) Principal component (PC) analysis plot of normalized counts of control and TE^sub^ MSCs. B) Heatmap of the relative expression values of the top 100 differentially expressed genes on tropoelastin. Asterisks denote genes in cell cycle and cell senescence pathways identified by Kyoto Encyclopedia of Genes and Genomes (KEGG) pathway enrichment analysis and cell cycle, mitotic, and aging pathways identified by Metascape pathway analysis. C) KEGG pathway enrichment for tropoelastin‐upregulated and D) downregulated genes. Confidence of pathway enrichment with the up or downregulated genes is represented by −log10(false discovery rate; FDR), and the number of genes (N. of Genes) represents the number of differentially expressed genes identified within each pathway. E) Volcano plot showing differentially expressed genes on tropoelastin, highlighting changes in cell cycle and senescence‐associated genes from KEGG and Metascape analyses. Red represents upregulated genes; blue, downregulated genes; and black, non‐differentially expressed genes. The horizontal dotted line represents the adjusted *p*‐value cut‐off (<0.05).

Bulk RNA sequencing revealed significant changes to the MSC transcriptome, following cell culture on substrate‐bound tropoelastin (TE^sub^) compared to tissue culture plastic (TCP) over 4 days. Principle component analysis (PCA) demonstrated that gene expression differences between control and TE^sub^ conditions, represented by PC1, are greater than the differences within each group, represented by PC2 (**Figure** **1**A). Differential gene expression analysis performed using DESeq2 identified 188 genes that were significantly upregulated with TE^sub^, and 143 genes that were significantly downregulated (Table S1, Supporting Information). The relative expression levels of the top 50 upregulated and downregulated genes are shown in the heatmap (Figure 1B). Kyoto Encyclopedia of Genes and Genomes (KEGG) pathway enrichment of these differentially expressed genes highlighted an increase in genes involved in the cell cycle, ECM‐receptor interactions, and the p53 pathway, which is crucial for regulating cell cycle progression, senescence, and DNA repair (Figure 1C). Enrichment for cancer‐related pathways (e.g., “microRNAs in cancer” and “transcriptional misregulation in cancer”) was predominantly due to the prominence of proliferative genes in these pathways. Similarly, the upregulated “progesterone‐mediated oocyte maturation” pathway comprises key genes implicated in the cell cycle pathway, such as CCNB1 and BUB1. Metascape and Gene ontology (GO) analyses also showed that TE^sub^‐upregulated genes were enriched for cell cycle‐related terms (Figure S1, Supporting Information).

Unexpectedly, in parallel with the mitogenic stimulation by TE^sub^, several downregulated genes were functionally mapped by KEGG to the cellular senescence pathway (CDKN1A (p21), MRAS, MAP2K3, CCND1, TGFB2, TGFBR1) and the senescence‐implicated TGF‐*β* signaling pathway (Figure 1D,E). Similarly, Metascape and GO analyses showed that downregulated genes were enriched not only for anti‐proliferative but also for aging pathways (Figure S1, Supporting Information). Taken together, these results emphasize the dualistic proliferative and anti‐aging responses to tropoelastin by MSCs.

### 2.2 Tropoelastin is Downregulated in Aged Cells and Exogenous Supplementation Extends the Replicative Lifespan of MSCs

**Figure 2:**
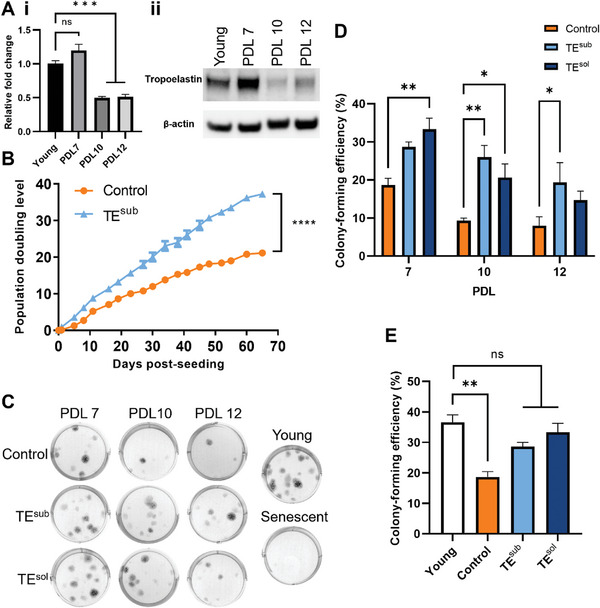
Tropoelastin enhances the replicative capacity of MSCs. A) Tropoelastin i) mRNA and ii) protein abundance decreases in MSCs with increasing population doubling level (PDL). Beta‐actin was included as a loading control. B) Population doubling kinetics of MSCs cultured with and without a tropoelastin substrate (TE^sub^). C) Representative images of colonies formed by control, TE^sub^, and soluble tropoelastin (TE^sol^) MSCs at different replicative ages. D) Comparative clonogenic abilities of variably‐cultured MSCs after 7, 10, and 12 PDLs. E) Colony‐forming efficiency of PDL 7 control and tropoelastin‐cultured MSCs compared to young PDL 4 MSCs. Experiments were performed with three replicates. ns, not significant; * *P* < 0.05; ** *P* < 0.01; *** *P* < 0.001; **** *P* < 0.0001.

Consistent with elastin loss in aging tissues, tropoelastin mRNA and protein expression in MSCs decreased with replicative aging (**Figure** **2**A). To determine the effect of tropoelastin on MSC replicative capacity, cells were cultured on TCP or on TE^sub^ until proliferative arrest, a defining hallmark of cellular senescence (Figure 2B). MSCs on TCP underwent a maximum of 21 population doublings before growth cessation, while cells on TE^sub^ reached their replicative limit only after 37 population doublings, indicating the ability of tropoelastin to delay replicative exhaustion and significantly extend the MSC proliferative lifespan by 76 ± 4% on average.

To investigate the functional modes of tropoelastin in regulating replicative senescence, MSCs were cultured either on TE^sub^ in standard growth media, or on TCP in growth media supplemented with soluble tropoelastin (TE^sol^). An aim was to observe if tropoelastin, a classical substrate component, exerts similar regulatory effects as a molecule in solution. Continuous culture of another independent MSC line supplemented with either TE^sub^ or TE^sol^ similarly increased the MSC proliferative limit by 53% and 60%, respectively, compared to control cells (Figure S2A, Supporting Information). These findings demonstrate that long‐term exposure to tropoelastin, via either cell anchorage to the substrate‐bound protein, or interactions with its soluble form, lengthens the proliferative lifespan of MSCs.

### 2.3 Tropoelastin Preserves the Clonogenic Capacity of Aging MSCs

To determine the effects of tropoelastin on the age‐associated cellular manifestation of senescent properties, MSC populations serially replicated under control, TE^sub^, or TE^sol^ conditions were banked at various population doubling levels (PDLs; 7, 10, and 12) and phenotypically and functionally characterized.

PDL‐matched MSCs grown with or without tropoelastin were assessed at different replicative ages for clonogenic efficiency in standard media (Figure 2C). As expected, the colony‐forming potential of all MSCs decreased with the increasing population doubling level, regardless of culture condition, demonstrating that clonogenic capacity is inversely correlated with replicative aging (Figure S2B, Supporting Information). Among age‐matched populations, TE^sub^ or TE^sol^ MSCs exhibited consistently higher clonogenic potential compared to control cells. TE^sub^ and TE^sol^ MSCs exhibited a 54 ± 7% and 78 ± 15% increase at PDL 7; 179 ± 33% and 121 ± 38% increase at PDL 10; and 142 ± 65% and 83 ± 30% increase at PDL 12, respectively (Figure 2D). Furthermore, while control cells showed an early 49 ± 5% decrease in colony‐forming efficiency at PDL 7 compared to the young (i.e., PDL 4) cells from which they were subcultured, this function was preserved in MSCs grown in tropoelastin‐supplemented conditions (Figure 2E).

### 2.4 Tropoelastin Suppresses SA *β*‐Gal Activity in Aging MSCs

**Figure 3:**
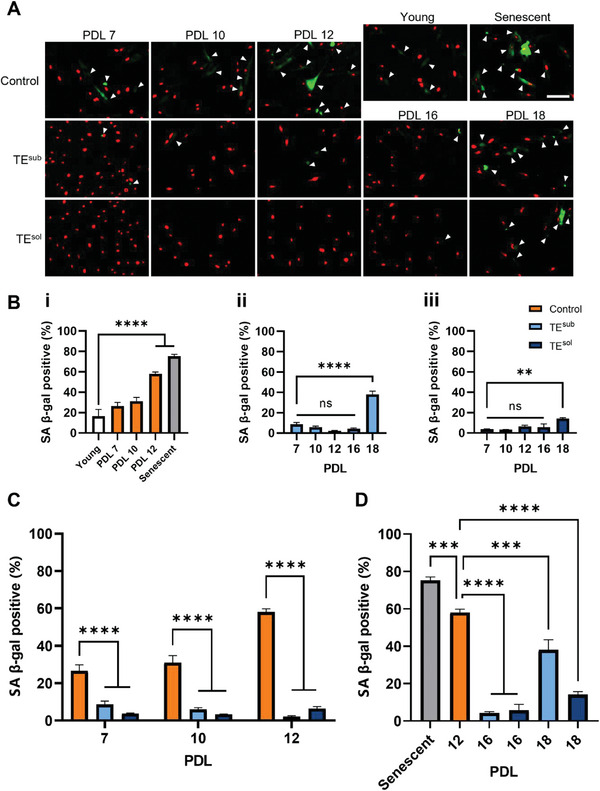
Tropoelastin reduces senescence‐associated beta‐galactosidase (SA *β*‐gal) activity in aging MSCs. A) Representative images of MSCs at different PDLs cultured with or without substrate‐bound or soluble tropoelastin. Cells were stained for SA *β*‐gal levels (green) and nuclei were stained with propidium iodide (red). White arrowheads point to SA *β‐*gal‐positive cells. Scale bar = 100 µm. B) SA *β*‐gal levels of i) control ii) TE^sub^, iii) TE^sol^ MSCs at increasing replicative ages. C) SA *β‐*gal activities of age‐matched control and tropoelastin‐grown MSCs. D) SA *β*‐gal activity of PDL 12 control MSCs compared to PDL 16/18 tropoelastin‐cultured MSCs and senescent MSCs. Experiments were performed with three replicates. ns, not significant; *** *P *< 0.001; **** *P *< 0.0001.

SA *β*‐gal activity is a well‐established marker of cellular senescence, with levels of SA *β*‐gal staining increasing with replicative age (**Figure** **3**A). Compared to young MSCs, the proportion of SA *β*‐gal positive cells significantly increased in control populations by PDL 12 (Figure 3Bi). In contrast, SA *β*‐gal positive cells remained minimally low in tropoelastin‐cultured populations, with an increase over young populations appearing only at PDL 18 (Figure 3Bii,iii). Between age‐matched populations across PDLs 7 to 12, SA *β*‐gal activity was consistently and significantly suppressed for MSCs treated with tropoelastin (Figure 3C). At PDL 12, TE^sub^ and TE^sol^ MSCs showed 96 ± 1% and 89 ± 2% reduction in SA *β*‐gal activity compared to control cells. Strikingly, the fraction of SA *β*‐gal positive cells was still significantly lower in tropoelastin‐grown populations at PDLs 16 and 18, compared to control cells at PDL 12 (Figure 3D). These results indicate that tropoelastin markedly delays the onset of SA *β‐*gal activity in MSCs during replicative aging.

### 2.5 Tropoelastin Maintains Normal Cell Size in Aging MSCs

**Figure 4:**
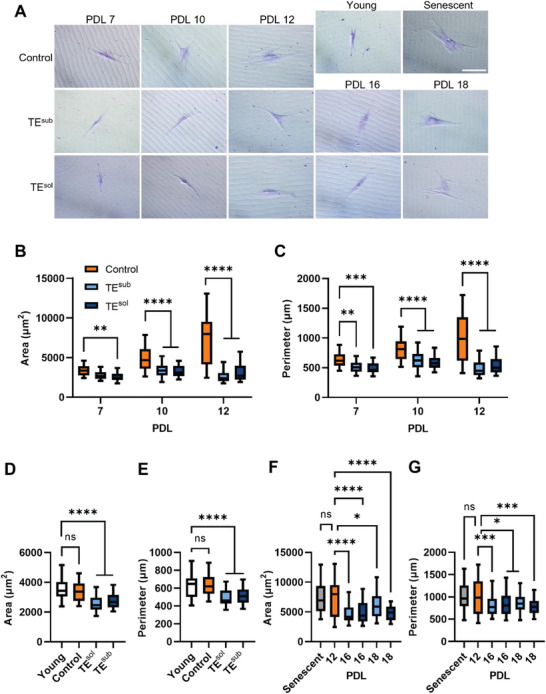
Tropoelastin preserves MSC size and shape during replicative aging. A) Representative images of control, TE^sub^, and TE^sol^ MSCs stained with crystal violet at different PDLs. Scale bar = 100 µm. Cell B) area and C) perimeter of age‐matched control, TE^sub^, TE^sol^ MSCs at PDLs 7, 10, and 12. Cell D) area and E) perimeter of PDL 7 control and tropoelastin‐cultured MSCs compared to young MSCs. Cell F) area and G) perimeter of senescent MSCs and PDL 16/18 tropoelastin‐cultured MSCs, compared to PDL 12 control cells. Measurements were conducted on fifty cells. ns, not significant; * *P *< 0.05; ** *P *< 0.01; *** *P *< 0.001; **** *P* < 0.0001.

MSCs grown in the variable presence of tropoelastin were further examined for phenotypic markers of senescence, such as cell size and morphology (**Figure** **4**A). Cell sizes ranged between 2400–13 000 µm^2^, consistent with previously reported sizes of human MSCs.^[^ ^26^, ^27^ ^]^ Indeed, cell size expectedly increased with age across all culture conditions, as measured by changes in cell area and perimeter (Figure S3A,B, Supporting Information). Among replicative age‐matched populations, both TE^sub^ and TE^sol^ MSCs displayed reduced cell area and perimeter at PDLs 10 and 12 compared to control cells, demonstrating tropoelastin‐mediated amelioration of another cellular senescent feature (Figure 4B,C). At PDL 12, TE^sub^ and TE^sol^ MSCs were 64 ± 1% and 58 ± 2% smaller in cell area compared to controls. Cell size measured by flow cytometry similarly revealed a 29% reduction in forward scatter area in PDL 10 TE^sol^ MSCs (Figure S3C, Supporting Information). In addition, cell morphological variability was smaller in tropoelastin‐cultured MSCs compared to control cells, suggesting that tropoelastin may also promote phenotypic homogeneity in aging populations.

Intriguingly, at PDL 7, TE^sub^ and TE^sol^ cells were smaller not only compared to control cells but also compared to the young cells from which they originated (Figure 4D,E). Consistent with the reduction in cell size, tropoelastin reduces the protein expression of mTOR (Figure S3D, Supporting Information). Furthermore, while control cells at PDL 12 were morphologically indistinguishable from senescent MSCs, cells grown with tropoelastin until PDL 18 persistently maintained a smaller area and perimeter, phenotypically indicating a lower level of senescence (Figure 4F,G).

### 2.6 Tropoelastin Modulates Molecular Markers of Senescence

**Figure 5:**
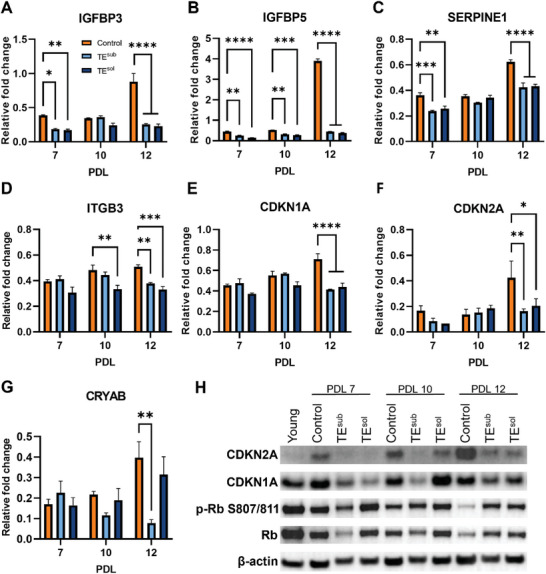
Molecular markers of senescence modulated by tropoelastin. Transcript levels of A) IGFBP3, B) IGFBP5, C) SERPINE1, D) ITGB3, E) CDKN1A, F) CDKN2A, and G) CRYAB in PDL‐matched control, TE^sub^, and TE^sol^ MSCs at different replicative ages. H) Protein abundance of CDKN2A, CDKN1A, phospho‐retinoblastoma (p‐Rb) S807/811, and retinoblastoma protein (Rb) in PDL‐matched control, TE^sub^, and TE^sol^ MSCs. Beta‐actin was included as a loading control. Experiments were conducted with three replicates. * *P *< 0.05; ** *P *< 0.01; *** *P *< 0.001; **** *P *< 0.0001.

To investigate whether the phenotypic preservation in tropoelastin‐cultured MSCs is accompanied by molecular changes, the expression of senescence‐associated genes was analyzed via qPCR. Expression of senescence markers IGFBP3, IGFBP5, SERPINE1, ITGB3, CDKN1A, CDKN2A (p16), and CRYAB were all validated to increase with increasing replicative age (Figure S4, Supporting Information). Bulk RNA‐seq had previously revealed early upregulation of the SASP component IGFBP3 and downregulation of IGFBP5 (Table S1, Supporting Information). Both markers and their regulator SERPINE1 were downregulated in tropoelastin‐expanded samples as early as PDL 7 and persisted until PDL 12 (**Figure** **5**A–C). At PDL 12, TE^sub^ and TE^sol^ MSCs exhibited a 71 ± 1% and 74 ± 4% decrease in IGFBP3 expression; 89 ± 0.2% and 91 ± 1% decrease in IGFBP5; and 32 ± 1% and 31 ± 3% decrease in SERPINE1, respectively. Although the tropoelastin receptor ITGB3 was not seen as significantly altered after early exposure as shown by RNA‐seq (Table S1, Supporting Information), tropoelastin‐mediated downregulation of ITGB3 was observed at later PDLs of 10 and 12 (Figure 5D). In line with an early decrease in the canonical senescence marker CDKN1A in RNA‐seq data (Figure 1E), CDKN1A expression decreased by 42 ± 1% and 38 ± 5%, and that of the related CDNK2A was reduced by 62 ± 4% and 51 ± 13%, in PDL 12 TE^sub^ and TE^sol^ samples, respectively, compared to control cells. As one of the most downregulated genes following early exposure to TE^sub^ (Figure 1E), CRYAB expression was reduced in TE^sub^ MSCs by 80 ± 4% at PDL 12 (Figure 5G), while TE^sol^ did not have an effect on CRYAB expression.

The expression of senescence markers was also examined at the protein level (Figure 5H). Consistent with the transcriptomics analyses, TE^sub^ and TE^sol^ MSCs showed consistently decreased levels of the cell cycle inhibitor CDKN2A compared to control cells, across PDLs 7, 10, and 12. Levels of CDKN1A also appeared to be downregulated in tropoelastin‐cultured cells at PDLs 7 and 12. Furthermore, the retinoblastoma protein (Rb) and its active phosphorylated form (p‐Rb), which are negatively regulated by CDKN2A, were found to be more abundant in TE^sub^ and TE^sol^ MSCs compared to their PDL‐matched control cells at PDL 12. These findings confirm that tropoelastin delays the expression and modulates the activity of senescence‐associated molecular markers.

### 2.7 Tropoelastin Reduces Senescence‐Associated Secretory Phenotype and Promotes Youth Markers in Aging MSCs

**Figure 6:**
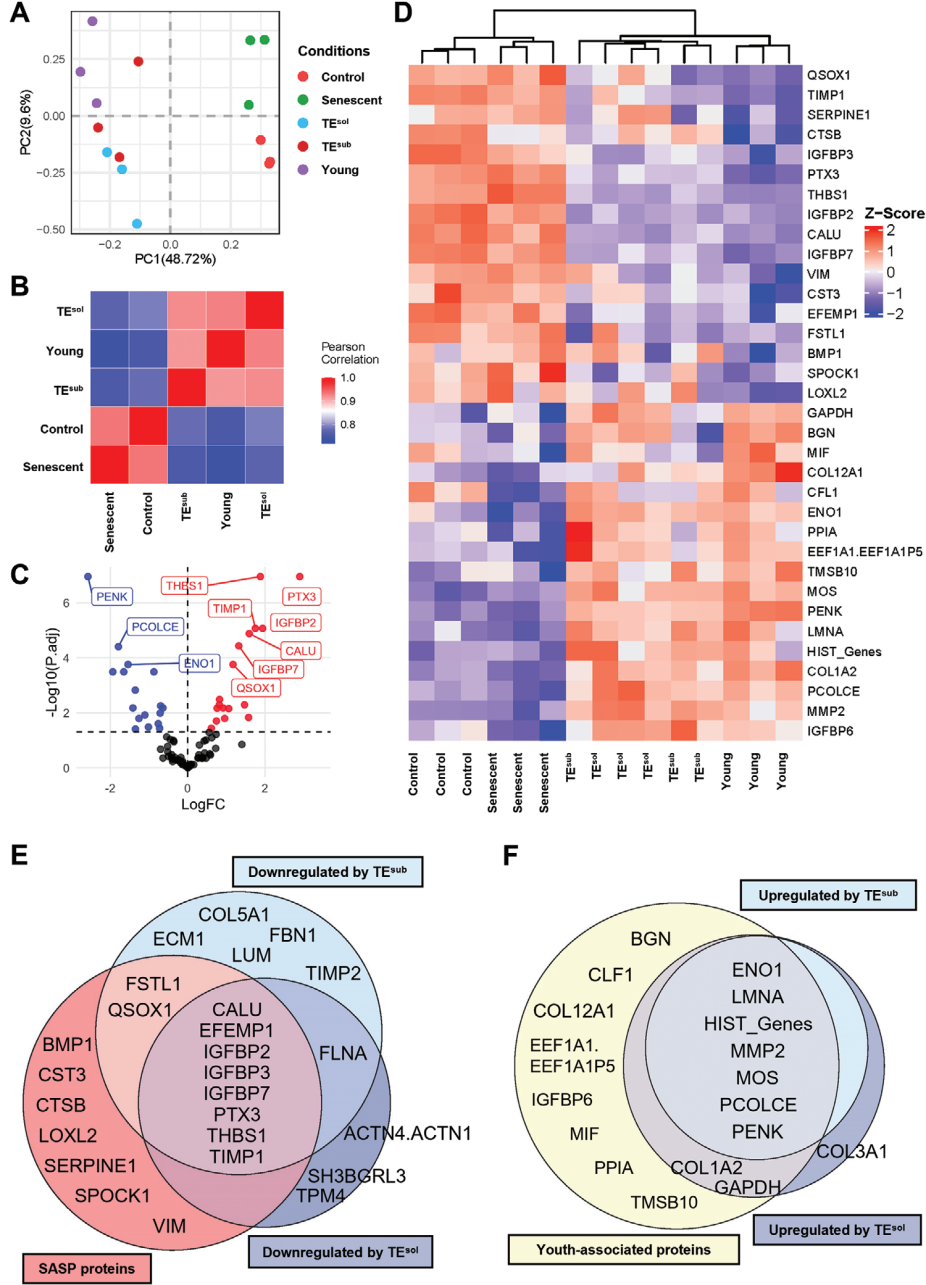
Secretome analysis of control and tropoelastin‐expanded MSCs at PDL 12. A) Principal component and B) Pearson correlation analysis of the secretome of PDL 12 control, TE^sub^, and TE^sol^ MSCs, compared to young and senescent cells. C) Volcano plot of differentially secreted proteins by senescent MSCs compared to young cells. Downregulated proteins are in blue, upregulated proteins are in red, and unchanged proteins are in black. The top 10 proteins with the most significantly altered abundance are labeled. The horizontal line represents the significance level (p‐adjusted value of 0.05). D) Heat map showing the comparative levels of differentially secreted proteins from Panel C in young, senescent, and PDL 12 control and tropoelastin‐grown MSCs. E) SASP proteins, which were upregulated in senescent MSCs compared to young cells, overlap with proteins significantly downregulated by substrate or soluble tropoelastin. F) Youth‐associated proteins, which were upregulated in young MSCs compared to senescent cells, overlap with proteins significantly upregulated by tropoelastin‐expanded MSCs over control cells.

Conditioned media from control, TE^sub^, and TE^sol^ MSCs at PDL 12 were analyzed via quantitative proteomics, and compared with the secretome of young and senescent cells. At the total secretome level, TE^sub^ and TE^sol^ MSCs more closely matched the secretory profile of young cells, while control cells more resembled senescent MSCs, as determined by PCA and Pearson correlation analysis (**Figure** **6**A,B).

Of the 83 proteins detected across all samples, 34 proteins were differentially expressed between young and senescent cells (Figure 6C). Within this age‐dependent secretome, 17 were upregulated in young cells, which were defined as youth‐associated proteins, and 17 were upregulated in senescent cells, which were defined as SASP proteins. These protein categories were then applied as the basis of comparison between age‐matched control and tropoelastin‐expanded MSC populations (Figure 6D). The majority of SASP proteins that were abundantly expressed by senescent MSCs were significantly downregulated in TE^sub^ and TE^sol^ cells compared to control cells. Conversely, most youth‐associated proteins that were highly secreted by young MSCs were also significantly upregulated in tropoelastin‐cultured cells compared to control cells. These findings highlight that while the age‐dependent secretome of control MSCs at PDL 12 reflected that of senescent cells, tropoelastin‐expanded MSCs markedly retained a youth‐like secretory profile.

The functional composition of the differentially expressed secretome between control and tropoelastin‐cultured MSCs was further examined (Figure 6E,F). Tropoelastin downregulated the secretion of aging‐associated ECM modifiers such as PTX3, QSOX1, THBS1, and TIMP1, in addition to matrix components such as ECM1, COL5A1, LUM, FBN1, and FSTL1, which have previously been demonstrated to be SASP components (Tables S2–S5, Supporting Information). In parallel, tropoelastin upregulated the ECM remodeling protein, matrix metallopeptidase 2 (MMP2). Downregulation of SASP components IGFBP2 and THBS1 was seen as an early effect in RNA‐seq (Table S1, Supporting Information), and was consistent with the protein downregulation observed in PDL 12 tropoelastin‐cultured MSCs. ECM1, IGFBP3, and EFEMP1 were found to be significantly upregulated by TE^sub^ in RNA‐seq, but these factors were ultimately decreased by tropoelastin in PDL 12 cells. Together, these results suggest that tropoelastin addition in culture partly dampens senescence‐associated changes in matrix components during long‐term culture. In addition to matrix components, tropoelastin also downregulated pro‐inflammatory IGFBPs whilst upregulating anti‐inflammatory PENK, which were similarly observed in young MSCs compared to senescent cells.

A predicted effect of the differential SASP expression between control and tropoelastin‐expanded MSCs is a difference in adipogenic potential. The increased levels of inflammatory SASP factors,^[^ ^28^ ^]^ as well as IGFBP2,^[^ ^29^ ^]^ IGFBP3,^[^ ^30^ ^]^ and TIMP1^[^ ^31^ ^]^ in senescing cells are expected to decrease adipogenic capability. Consistent with this prediction, adipogenic differentiation of MSCs declined with replicative age (Figure S5A, Supporting Information). In contrast, TE^sub^ and TE^sol^ cells, which secrete reduced SASP proteins and increased pro‐adipogenic MMP2,^[^ ^32^ ^]^ retained significantly higher adipogenic capacity compared to age‐matched controls (Figure S5B, Supporting Information).

## 3 Discussion

**Figure 7:**
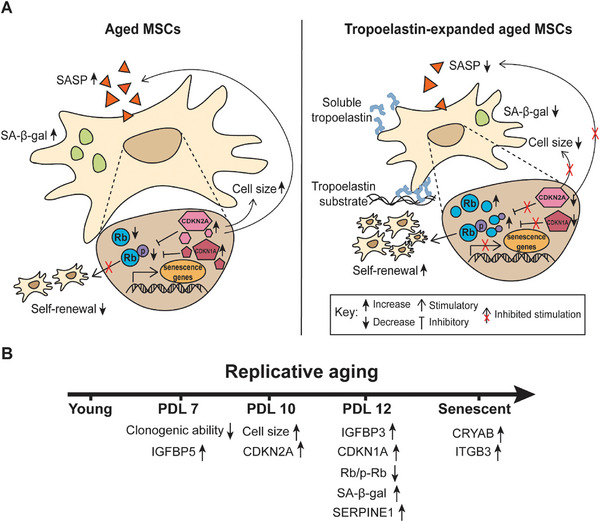
Cellular senescence markers in MSCs. A) MSC senescence markers that are modulated by tropoelastin. B) MSC senescence markers appear at various replicative ages along the progression of cellular senescence.

Stem cell senescence is a crucial component in age‐related disease due to the role stem cells play in tissue renewal and maintenance.^[^ ^33^ ^]^ For example, senescing MSCs unable to maintain bone tissue homeostasis are thought to lead to osteoporosis,^[^ ^34^ ^]^ which is among the most prevalent degenerative diseases in the elderly population.^[^ ^35^ ^]^ Following in vitro MSC expansion, senescence‐associated decline in therapeutic functions remains one of the biggest hurdles of stem cell therapies.^[^ ^9^ ^]^ While the ability of the ECM to regulate MSC functions is well‐documented, few studies have explored its role in driving and maintaining age‐related cellular fitness. Incidentally, aging tissues are characterized by ECM dysregulation, including a decline in elastin production and progressive elastin loss in the matrix.^[^ ^25^, ^36^ ^]^ This work demonstrates the role of the elastin monomer, tropoelastin, in modulating MSC replicative senescence (**Figure** **7**A).

Tropoelastin triggers a strong mitogenic signal capable of promoting MSC proliferation,^[^ ^20^ ^]^ consistent with the dominant upregulation of cell cycle genes and concomitant downregulation of cell cycle inhibitors observed in bulk transcriptomic profiles of control and tropoelastin‐treated MSCs. Sustained stimulation of mitogenic pathways has been reported to exert DNA replicative stress and underpin senescent features including proliferative arrest and SASP.^[^ ^37^, ^38^, ^39^, ^40^ ^]^ Surprisingly, however, genes correlated to senescence onset and progression were also downregulated in tropoelastin‐cultured MSCs compared to control cells, implicating the unique, dualistic roles of tropoelastin in promoting proliferation while delaying cellular aging.

Accordingly, MSCs expanded either on a tropoelastin substrate or in tropoelastin‐supplemented media not only proliferated more rapidly but also underwent 16 further population doublings compared to control cells, which equates to the generation of 65 000‐fold more cells, before proliferative arrest. Self‐renewal is a defining characteristic of stem cells implicated in maintaining tissue homeostasis in vivo and supporting stem cell therapies in vitro.^[^ ^41^ ^]^ This ability of tropoelastin to extend MSC replicative lifespan supersedes that of media supplements such as FGF2, PDGF‐BB, and ascorbic acid, which have been documented to support ten or more population doublings.^[^ ^14^ ^]^ However, the cell phenotypic or functional benefits of such supplements are shown to reverse at late stages of growth. A potential confounding factor is that such studies have been performed on populations matched at passage level but not at replicative age, resulting in comparisons between treated cells that are more than double the replicative age of control cells.^[^ ^14^, ^42^ ^]^ In this study, age‐matched control and tropoelastin‐cultured MSC populations were systematically compared for markers of senescence to determine the extent and efficacy of tropoelastin‐mediated seno‐protection. The comparisons of MSCs at the same PDL are based on the notion that PDL is a more accurate parameter of replicative age and a superior measure of replicative lifespan.^[^ ^43^, ^44^ ^]^

Consistent with their extended replicative capacity, MSCs expanded in the presence of tropoelastin demonstrated increased preservation of clonogenic capacity compared to PDL‐matched controls, which persisted even at late replicative ages. Colony‐forming unit ability is predictive of MSC multipotency and clinical outcomes after transplantation,^[^ ^45^, ^46^ ^]^ indicating the potential of tropoelastin to preserve key MSC regenerative properties, including self‐renewal, in the long term. Incidentally, tropoelastin‐expanded MSCs still displayed gradual clonogenic exhaustion, indicating that tropoelastin delays or slows senescence progression, but does not eliminate senescence induction.

Quantification of SA *β‐*gal activity, a highly established senescence marker, revealed a dramatic reduction of this senescent phenotype by tropoelastin. A high level of SA *β*‐gal activity measured at near‐neutral pH represents over‐production of the lysosomal beta‐galactosidase,^[^ ^47^ ^]^ as well as increased lysosomal content and autophagic activity,^[^ ^48^, ^49^ ^]^ associated with replicatively senescent MSCs.^[^ ^50^ ^]^ Lysosomal autophagy has been shown to be suppressed by protein kinase B (AKT),^[^ ^51^, ^52^ ^]^ which tropoelastin is known to activate by integrin signaling.^[^ ^20^ ^]^ Thus, tropoelastin‐mediated reduction of SA *β*‐gal activity during MSC aging may arise from a dampening of excess autophagic activity via AKT activation. Interestingly, SA *β‐*gal levels did not significantly increase in aging MSCs until growth arrest was apparent, which occurred at PDL 12 for control MSCs and PDL 18 for TE^sub^ and TE^sol^ cells. This finding suggests that SA *β*‐gal staining does not detect the basal levels of autophagy required for stemness,^[^ ^53^ ^]^ but instead reflects the aging‐driven enhanced degradation of proteins such as SIRT1, KEAP1, and TNIP, proteolysis of which contribute to the establishment of senescence and maintenance of cell viability during senescence.^[^ ^54^, ^55^ ^]^

Tropoelastin‐cultured MSCs also showed a reduction in cell size across different replicative ages. Cell morphological changes are another hallmark of replicative senescence in MSCs.^[^ ^56^, ^57^ ^]^ In addition, independent of donor age, MSC size is inversely correlated with function,^[^ ^58^, ^59^ ^]^ and consequently has been proposed as a marker for the isolation of senescent MSCs.^[^ ^60^ ^]^ Indeed, the smaller morphological features of tropoelastin‐expanded cells concurred with their higher clonogenic potential, compared to control cells at the same replicative age. While a small decrease in cell size was further noted for tropoelastin‐cultured cells at PDL 7 compared to young PDL4 cells, this difference was not accompanied by an increase in colony‐forming efficiency.

Cell mass increase is normally tightly coupled to cell division rates to maintain macromolecule homeostasis.^[^ ^61^, ^62^ ^]^ However, checkpoint activation delays can arrest the cell cycle and enlarge cell size.^[^ ^63^ ^]^ The resulting dilution of cytoplasmic content can, in turn, trigger senescence,^[^ ^64^, ^65^ ^]^ suggesting a positive feedback loop between cell size increase and senescence. Accordingly, the smaller‐sized tropoelastin‐cultured MSCs expressed lower levels of cell cycle inhibitors CDKN1A and CDKN2A compared to age‐matched controls. It is possible that tropoelastin promotion of the cell cycle prevents cell enlargement, and thereby inhibits the amplifying effects of increased cell size on senescence. Under the control of AKT, mTOR regulates cell size and increases CDKN1A expression.^[^ ^63^, ^66^ ^]^ Tropoelastin is known to activate AKT,^[^ ^20^ ^]^ and supplementation in either forms lower mTOR expression. We postulate that mTOR may mediate tropoelastin‐based reduction of MSC senescent phenotypes. In addition, cell morphological variability appears to increase with PDL, most prominently in control cells at PDL 12, which reflects the typical spectrum of heterogeneity in senescent populations.^[^ ^6^ ^]^ In contrast, the smaller range of heterogeneity in tropoelastin‐expanded cells suggests that tropoelastin not only reduces average population senescence but also promotes uniform fitness of individual cells within the population.

The senescent phenotypes of aging MSCs were accompanied by increased expression of senescence‐associated genes. Accordingly, tropoelastin downregulated the expression of these senescence‐associated genes, corresponding to the subdued senescent phenotypes of tropoelastin‐expanded cells. IGFBP3 and IGFBP5 have been shown to significantly increase in senescent MSCs and exert pro‐senescence effects via paracrine actions,^[^ ^67^, ^68^ ^]^ while SERPINE1 inhibits protease breakdown of the IGFBPs and enhances their stimulation.^[^ ^69^, ^70^ ^]^ The IGFBPs are additionally known to increase the expression of senescence‐inducing cell‐cycle inhibitors such as CDKN1A.^[^ ^71^, ^72^ ^]^ Furthermore, tropoelastin downregulated ITGB3 expression, which increases in aging cells and enhances senescence by upregulating TGF‐*β* and CDKN1A pathways.^[^ ^73^ ^]^ Accordingly, RNAseq revealed the downregulation of TGF*‐β* and CDKN1A pathways by tropoelastin. Therefore, the seno‐protective effects of tropoelastin may also be attributed to its downregulation of IGFBP3, IGFBP5, and SERPINE1 genes as early as PDL 7, as well as ITGB3 from PDL 10.

Age‐dependent expression was more pronounced with CDKN2A compared to CDKN1A, which agrees with previous reports of CDKN2A as a more robust marker of replicative aging than CDKN1A in MSCs.^[^ ^74^ ^]^ Both CDKN2A and CDKN1A inhibit the cell cycle,^[^ ^75^, ^76^ ^]^ by complexing with cyclin‐dependent kinase (CDK) 4/6,^[^ ^77^ ^]^ which, in turn, induces hypophosphorylation of the Rb target at S807/811and inhibits the stimulatory effect of p‐Rb on cell proliferation.^[^ ^78^, ^79^ ^]^ Consistent with this pathway, CDKN2A and CDKN1A upregulation in senescing control MSCs was associated with reduced levels of p‐Rb S807/811. In contrast, tropoelastin represses age‐dependent upregulation of CDKN2A and CDKN1A and increases p‐Rb availability to maintain proliferative capacity. (Incidentally, protein levels of senescence markers CDKN1A and MAP2K3 were not altered by short‐term tropoelastin supplementation (Figure S6, Supporting Information), despite early transcriptomic downregulation in the RNA‐seq results.) Tropoelastin modulation of these cell cycle proteins is the likely mechanism underpinning its ability to protect against senescence‐associated proliferative arrest.

Furthermore, there is a negative correlation between total Rb protein levels and MSC senescence. There have been variable findings on the link between Rb and senescence, which may depend on the cell type. In fibroblasts, Rb is thought to induce senescence‐associated heterochromatin and proliferative arrest.^[^ ^80^, ^81^ ^]^ In contrast, and consistent with our results, Rb is shown to decrease in senescing MSCs,^[^ ^82^ ^]^ leading to the downregulation of DNMT1 and loss of DNMT1 inhibition of CDKN2A and CDKN1A. The resulting increase in CDKN2A further suppresses transcription of Rb,^[^ ^83^ ^]^ amplifying senescence progression. In contrast, tropoelastin‐expanded PDL 12 cells retained higher Rb levels and reduced CDKN2A, which has been shown to extend replicative lifespan.^[^ ^81^ ^]^

Consistent with its effects on other markers of senescence, tropoelastin also protects against senescence‐associated changes in the MSC secretome. Proteomic analysis revealed upregulation of CALU, EFEMP1, IGFBP2/3/7, PTX3, THBS1, and TIMP1 in senescent cells compared to young MSCs, and a corresponding increase in control cells compared to tropoelastin‐expanded cells. These factors have all been linked to SASP in previous studies (Table S3, Supporting Information). IGFBPs and THBS1 are pro‐inflammatory and exacerbate senescence through autocrine effects in MSC populations.^[^ ^84^ ^]^ In contrast, PENK, which is upregulated in young cells, is a potent anti‐inflammatory factor that plays a role in MSC‐mediated immunomodulation.^[^ ^85^ ^]^ Compared to age‐matched controls, tropoelastin‐expanded MSCs secrete decreased IGFBPs and THBS1, and increased PENK, which may help maintain MSC immunomodulatory function.^[^ ^8^ ^]^ This tropoelastin‐mediated decrease in SASP may be attributed to decreased CDKN2A levels,^[^ ^86^ ^]^ or since SASP develops over time,^[^ ^87^ ^]^ due to tropoelastin‐cultured cells reaching the same replicative age faster than control MSCs. Another potential mechanism by which SASP is dampened by tropoelastin is through the downregulation of the TGF‐*β* pathway (Figure 1D), which underpins the production of IGFBPs.^[^ ^88^, ^89^ ^]^

Prolonged exposure to SASP from senescent cells can trigger neighboring cells to undergo senescence, leading to tissue degeneration in vivo.^[^ ^3^ ^]^ This positive feedback loop is particularly detrimental for the therapeutic potential of in vitro cultured MSCs, due to the lack of an effective clearance system for senescent cells.^[^ ^28^ ^]^ Thus, the amelioration of SASP in aged MSCs by tropoelastin has the potential to improve the clinical translation of MSCs, for which in vitro expansion is necessary.

Proteomic profiling revealed that while the secretome of control cells at PDL 12 mirrors that of senescent cells, age‐matched tropoelastin‐cultured MSCs closely resemble that of young PDL 4 cells. Young and tropoelastin‐expanded MSCs showed similar upregulation of ENO1, LMNA, and MOS, which are canonical cytoplasmic or nuclear proteins previously identified in other secretome data sets and reported to have satellite extracellular functions.^[^ ^84^, ^90^, ^91^ ^]^ These cytoplasmic or nuclear proteins may also arise from extracellular vesicles that encapsulate various macromolecules.^[^ ^92^ ^]^

Cellular senescence is a spectrum, with phenotypic and functional changes presenting at varying stages throughout the aging trajectory. For example, while clonogenic potential declined between cells at PDL 4 and PDL7, cell morphological changes appeared only at PDL 10. SA *β‐*gal activity, the most commonly referenced senescence marker, did not increase until PDL 12 (Figure 7B). Therefore, assaying any single marker at a snapshot in time may not reliably inform on the senescence status of a population.

MSC exposure to tropoelastin over a replicative lifetime is demonstrated to consistently delay the onset of multiple senescence hallmarks. This strong seno‐protective role of tropoelastin is consistent with a correlation between elastin haploinsufficiency and an aging phenotype in animals and humans. For example, heterogeneous knockout of elastin in mice is shown to accelerate the manifestation of aging in mesodermal systems, such as the circulatory system and the kidney.^[^ ^93^, ^94^ ^]^ Similarly, patients with Williams–Beuren Syndrome, characterized by heterozygous deletion of the elastin gene, also exhibit early‐onset aging.^[^ ^95^, ^96^, ^97^ ^]^ Tropoelastin deficiency was also recently shown to induce premature senescence in fibroblasts.^[^ ^98^ ^]^

This study demonstrates for the first time that the ECM is a potent and active driver of MSC age‐related fitness. Tropoelastin expression is functionally associated with early development and tissue repair, periods in which rapid stem cell expansion and protection against aging stresses are dually beneficial. Progressive loss of this protein is a hallmark of tissue and cell aging. Our results demonstrate that exogenous supplementation of tropoelastin prolongs cell functional vigor, and delays the phenotypic changes and functional decline associated with senescence. While a study has shown that the supplementation of fibronectin rejuvenated muscle stem cells by altering cellular adherence,^[^ ^99^ ^]^ tropoelastin does not appear to exert influence over the short‐term adhesion efficiency of MSCs (Figure S7, Supporting Information), suggesting that tropoelastin‐mediated seno‐protection is not underpinned by changes to cell attachment capability. Understanding the stimuli that influence stem cell senescence bears significance for both in vitro cell manipulation and organismal aging. Our findings advocate for the incorporation of substrate or soluble tropoelastin into MSC culture platforms, as a straightforward strategy to enhance the production of cells that preserve regenerative capabilities with age. Furthermore, this study underscores the potential of the ECM as a new target in regenerative medicine interventions aimed at modulating cellular senescence for age‐related pathologies.

## 4 Experimental Section

All reagents were purchased from Sigma‐Aldrich unless indicated.

### MSC Culture

Human bone marrow‐derived MSCs (American Type Culture Collection) were cultured in standard media composed of alpha minimum essential medium supplemented with 10% MSC‐qualified fetal bovine serum (Gibco), 2 mm *L*‐glutamine, 100 units mL^−1^ penicillin and 100 µg mL^−1^ streptomycin. TE^sol^: MSCs were grown in standard media supplemented with 20 µg mL^−1^ recombinant wild‐type human tropoelastin (corresponding to GenBank entry AAC98394, residues 27–724), on TCP blocked with standard media at 4 °C overnight. TE^sub^: MSCs were cultured in standard media on TCP coated with 20 µg mL^−1^ tropoelastin in phosphate‐buffered saline (PBS) (10 mm sodium phosphate, 150 mm sodium chloride, pH 7.4) at 4 °C overnight.^[^ ^20^ ^]^ Senescent MSCs were defined as cells that have displayed a culture growth plateau for at least two weeks. Cultures were maintained at 37 °C in a 5% CO_2_ normoxic humidified incubator, and corresponding media were changed every 2–3 days.

When cultures reached 70–80% confluency, cells were washed with PBS and incubated with 0.05% (w/v) trypsin‐EDTA for 5 min at 37 °C. Detached cells were centrifuged at 270 × *g* and re‐seeded at 4000–6000 cells cm^−2^. At specific PDLs, cells were frozen in standard media with 10% (v/v) dimethyl sulfoxide.

Cells used in subsequent assays were thawed, resuspended in standard media, and pelleted by centrifugation at 270 × *g*, before plating in standard media on TCP to recover overnight.

### Bulk RNA Sequencing (RNA‐Seq)

MSCs grown on either TCP or tropoelastin‐coated TCP were harvested after 4 days, snap‐frozen in liquid nitrogen, and sent to the Australian Genome Research Facility for library preparation and RNA sequencing. RNA was extracted from the cell pellets (0.5 × 10^6^ cells) using the RNeasy Plus mini kit (Qiagen). Illumina NovaSeq RNA sequencing of 6 poly‐A RNA stranded samples was performed using 100 base pair single‐end runs. Per base sequence quality of all samples showed that more than 96% of bases exceeded the Q30 threshold. Reads were also screened for the presence of adapters and the Illumina Stranded mRNA Prep adapter sequence (CTGTCTCTTATACACATCT) was trimmed using Trim Galore. Cleaned reads were then aligned against the Homo Sapiens genome (HG38) and indexed with STAR (v2.5.3a). Counts were summarized at the gene level using featureCounts (v1.5.3) and normalized using variance stabilizing transformation for quality assessment. Differential gene expression analysis was then performed using DESeq2 (v1.32.0) with the default normalization method (library size adjustment, log transformation, regularization, weighted averaging, and median‐of ratios).^[^ ^100^ ^]^ Significance was defined as adjusted p values (padj) less than 0.05. KEGG and GO enrichment were performed on the significantly up and downregulated genes using ShinyGO (v0.80) with the default settings.^[^ ^101^ ^]^ Metascape (accessed on 8/03/2022) was also used for gene annotation to identify key biological pathways and processes. “Express analysis” was performed on up and downregulated gene lists separately.^[^ ^102^ ^]^

### Colony‐Forming Unit Assay

MSCs were seeded at 5 cells cm^−2^ in standard media. After 14 days, cells were fixed with 4% (v/v) formaldehyde in PBS for 20 min and subsequently stained with 0.1% (w/v) crystal violet in 0.2 m 2‐(N‐morpholino)ethanesulfonic acid (MES) buffer, pH 5.5, for 1 h. The excess stain was washed off with Milli‐Q water three times. Colonies were imaged with the ChemiDoc MP Imaging System, and manually counted.

### SA *β‐*Gal Staining

Cells were seeded at 5000 cells cm^−2^ and left to attach overnight. Media was aspirated from the wells and cells were washed with PBS. Cells were fixed with 2% (v/v) formaldehyde in PBS for 10 min at room temperature, then washed with 1% (w/v) bovine serum albumin in PBS. Cells were stained using a CellEvent Senescence Green Detection Kit (Thermo Fisher), incubated at 37 °C in the dark for 2 h, then imaged using the IncuCyte SX5 Live‐Cell Analysis System (Sartorius). To obtain the total cell count, cells were permeated with 0.3% (v/v) Triton X‐100 in PBS for 10 min at room temperature, stained with 10 µg mL^−1^ propidium iodide, and re‐imaged. Images were analyzed using FIJI software.^[^ ^103^ ^]^ SA *β‐*gal positive cells were expressed as a percentage of total cells.

### Cell Size Measurements

Cells were seeded at 10 cells cm^−2^ and left to attach overnight. Cells were fixed with 4% (v/v) formaldehyde for 20 min and subsequently stained with crystal violet for 1 h. The excess stain was washed off with Milli‐Q water three times. Fifty individual cells were imaged using an Axio Vert.A1 Inverted Microscope (Zeiss). Cell area and perimeter were measured using FIJI.

### Real‐Time PCR

Sample RNA was isolated using the PureLink RNA Isolation Kit (Thermo Fisher) according to the manufacturer's instructions, and reverse transcribed with the iScript cDNA Synthesis Kit (Bio‐Rad). Real‐time quantitative PCR analysis was performed with PowerUp SYBR Green master mix (Thermo Fisher) using a QuantStudio 7 Flex system (Thermo Fisher). KiCqStart SYBR primers were used to amplify each gene as per **Table** **1**. Target gene expression was normalized to beta‐actin (ACTB), and expressed as a fold change relative to senescent cells.

**Table 1:** Primer sequences for genes assayed via real‐time PCR.

Gene target	Forward primer (5′‐3′)	Reverse primer (5′‐3′)
ACTB	GACGACATGGAGAAAATCTG	ATGATCTGGGTCATCTTCTC
CRYAB	CAGAGGAACTCAAAGTTAAGG	ATGAAACCATGTTCATCCTG
ELN	TAAAGCAGCTAAATACGGTG	AGGAAGCTCATTTTCTCTTC
IGFBP3	AATCATCATCAAGAAAGGGC	GAACTTCAGGTGATTCAGTG
IGFBP5	AAGTCAAGATCGAGAGAGAC	TGGGTCAGCTTCTTTCTG
ITGB3	CTCCGGCCAGATGATTC	TCCTTCATGGAGTAAGACAG
CDKN1A	CAGCATGACAGATTTCTACC	CAGGGTATGTACATGAGGAG
CDKN2A	AGGTCCCTCAGACATCC	AATGAAAACTACGAAAGCGG
SERPINE1	CGCAACGTGGTTTTCTC	CATGCCCTTGTCATCAATC

### Western Blot

Cells were seeded at 5000 cells cm^−2^ and left to attach overnight. Cells were washed with PBS and lysed with RIPA buffer (Thermo Fisher) supplemented with Pierce protease and phosphatase inhibitor (Thermo Fisher). Cell debris was pelleted at 12 000 × *g* for 15 min, and protein concentration was estimated via a Pierce bicinchoninic acid assay (Thermo Fisher S). Total protein (5 µg) was denatured at 85 °C for 10 min, separated by 4–12% Bis‐Tris SDS‐PAGE (Thermo Fisher), then transferred to a polyvinylidene fluoride membrane (Millipore). The membrane was blocked with 5% (w/v) skim milk in Tris‐buffered saline with Tween 20 (TBST) (25 mM Tris‐HCl, pH 7.6, 140 mm NaCl, 0.1% Tween 20) for 1 h at room temperature and incubated with an affinity‐purified rabbit polyclonal anti‐elastin (1 in 1000, *α*‐hTE‐17‐F, which was a kind gift from Professor Dieter Reinhardt, McGill University) and beta‐actin (1 in 1000, Cell Signalling technology #4970) antibodies overnight. After three washes with TBST, the membranes were incubated with horseradish peroxidase (HRP)‐conjugated anti‐rabbit IgG antibody (1 in 5000, Cell Signalling Technology #7074) for 1 h at room temperature. Blots were washed three times with TBST, developed with Immobilon ECL Ultra Western HRP Substrate (Millipore), and imaged using the ChemiDoc MP Imaging System (Bio‐Rad).

For senescence markers, analytes were similarly prepared, and the membranes were incubated with CDKN2A (1 in 1000, Cell Signalling Technology #80772) or p‐RB Ser807/811 (1 in 1000, Cell Signalling Technology #8516) antibodies at 4 °C overnight. The blots were similarly developed and the membranes were stripped twice with mild stripping buffer (1.5% (w/v) glycine, 0.1% (w/v) sodium dodecyl sulfate, 1% (v/v) Tween 20), neutralized twice with PBS, washed twice with TBST, and incubated with CDKN1A (1 in 1000, Cell Signalling Technology #2947), beta‐actin, or Rb (1 in 2000, Cell Signalling Technology #9309) antibodies at 4 °C overnight. Protein levels were probed in a similar manner as described with HRP‐conjugated anti‐rabbit IgG antibody or anti‐mouse IgG antibody (1 in 5000, Cell Signalling Technology #7076).

### Secretome Analysis by Liquid Chromatography‐Tandem Mass Spectrometry (LC‐MS/MS)

Cells were seeded at 5000 cells cm^−2^ and left to attach overnight. The attached MSCs were washed twice with PBS, then incubated in alpha minimum essential medium without supplements for 24 h. Conditioned media were collected, and cell debris was pelleted at 15 000 × *g* for 10 min. Proteins were precipitated with 4 volumes of ice‐cold acetone overnight and pelleted at 10 000 × *g* for 20 min. The pellets were washed with ice‐cold acetone and air‐dried for 30 min. Proteins were resuspended in 6 M:4 m urea: thiourea with 10 mm dithiothreitol, sonicated for 2 min, incubated at room temperature for 1 h, followed by alkylation with 25 mm iodoacetamide at room temperature in darkness for 30 min. Samples were then diluted with five volumes of 50 mm ammonium bicarbonate, and 30 µg protein was digested with 1 µg trypsin (Promega) at 37 °C overnight at 600 rpm. Samples were then desalted and cleaned up using Oasis hydrophilic‐lipophilic‐balance cartridges (Waters), vacuum‐dried, and reconstituted in 100 µL of 3% (v/v) acetonitrile and 0.1% (v/v) formic acid prior to LC‐MS analysis. Each sample (2 µg) was separated by LC using a Nexera Series UHPLC LC‐40 system (Shimadzu) coupled to an Infinitylab poroshell 120 Å EC‐C18 2.1 mm × 50 mm, 1.9 µm column (Agilent) with an Infinitylab poroshell 120 Å EC‐C18 2.1 mm × 50 mm, 1.9 µm UHPLC Guard (Agilent). LC mobile phase buffers were comprised of A: 0.1% (v/v) formic acid, and B: 99.9% (v/v) acetonitrile with 0.1% (v/v) formic acid. Peptides were eluted using a linear gradient (0.8 µL min^−1^) of 9% B to 29% B over 5 min, then to 42% B for under 1 min, and to 80% B for 12 s, ending with an 80% B wash at 1 µL min^−1^ over 20 s. Mass spectra were acquired in the mass‐to‐charge ratio (*m/z*) range of 400–900 using a 7600 zenoSWATH Q‐TOF mass spectrometer (Sciex), and 60 varying SWATH fragmentation windows of 2–16 *m/z*, with varying collisional voltages of 19–43 V.

The raw SWATH data were analyzed with Spectronaut version 18.4.231017.55695 (Biognosys). The default BGS Factory settings were used for a library‐free DIA analysis using a directDIA+ and a protein database file as input. In brief, the data analysis was set to a trypsin digest, with modifications of carbamidomethyl (S), acetyl (protein N‐terminus), and oxidation (M). Interference correction on MS1 and MS2 levels was enabled. The false discovery rate (FDR) was set to 1% at peptide precursor and protein levels. The database used consisted of filtered and reviewed sequences within the Homo sapiens organism database (UniProt).

An ExpressionSet (Biobase 2.58.0) object was created to hold the intensities count matrix and the annotated metadata. “Na” values were omitted, and the counts were log‐transformed. Transformed values replaced the raw count matrix and subsequent PCA was performed to visualize variation within and between conditions. A linear model was fitted for each gene using the Limma (3.54.2) linear model and genes were ranked using the empirical Bayes method (eBayes). The Benjamini–Hochburg method was used to control for the false discovery rate. Differentially expressed genes were then identified and significance was defined as padj <0.05.

### Statistical Analyses

Statistical analyses were performed with Prism (v9.5.1). Data were presented as mean ± standard error of the mean with three independent experiments unless specified otherwise. Comparisons of means were made using analysis of variance with Dunnett or Tukey's corrections for multiple comparisons (ns, not significant; * *P *< 0.05; ** *P* < 0.01; *** *P* < 0.001; **** *P* < 0.0001).

## Conflict of Interest

A.W. is the Scientific Founder of Elastagen Pty Ltd, which was sold to Allergan, an AbbVie company. The other authors declare no conflict of interest.

## Supporting information

Supporting Information

## Data Availability

The data that support the findings of this study are available from the corresponding authors upon reasonable request.
