# Supplementary material for: The Matrix Protein Tropoelastin Prolongs Mesenchymal Stromal Cell Vitality and Delays Senescence During Replicative Aging
Source: Adv Sci (Weinh). 2024 Aug 9;11(39):2402168. doi: 10.1002/advs.202402168 (PMC11497112; doi:10.1002/advs.202402168)
Supplement: Supplementary file 1 — Supporting Information [file ADVS-11-2402168-s001.docx]

**Supporting Information**

**The Matrix Protein Tropoelastin Prolongs Mesenchymal Stromal Cell Vitality and Delays Senescence During Replicative Ageing**

*Sunny Shinchen Lee, Aleen Al Halawani, Jonathan D. Teo, Anthony S. Weiss*, and Giselle C. Yeo**

**
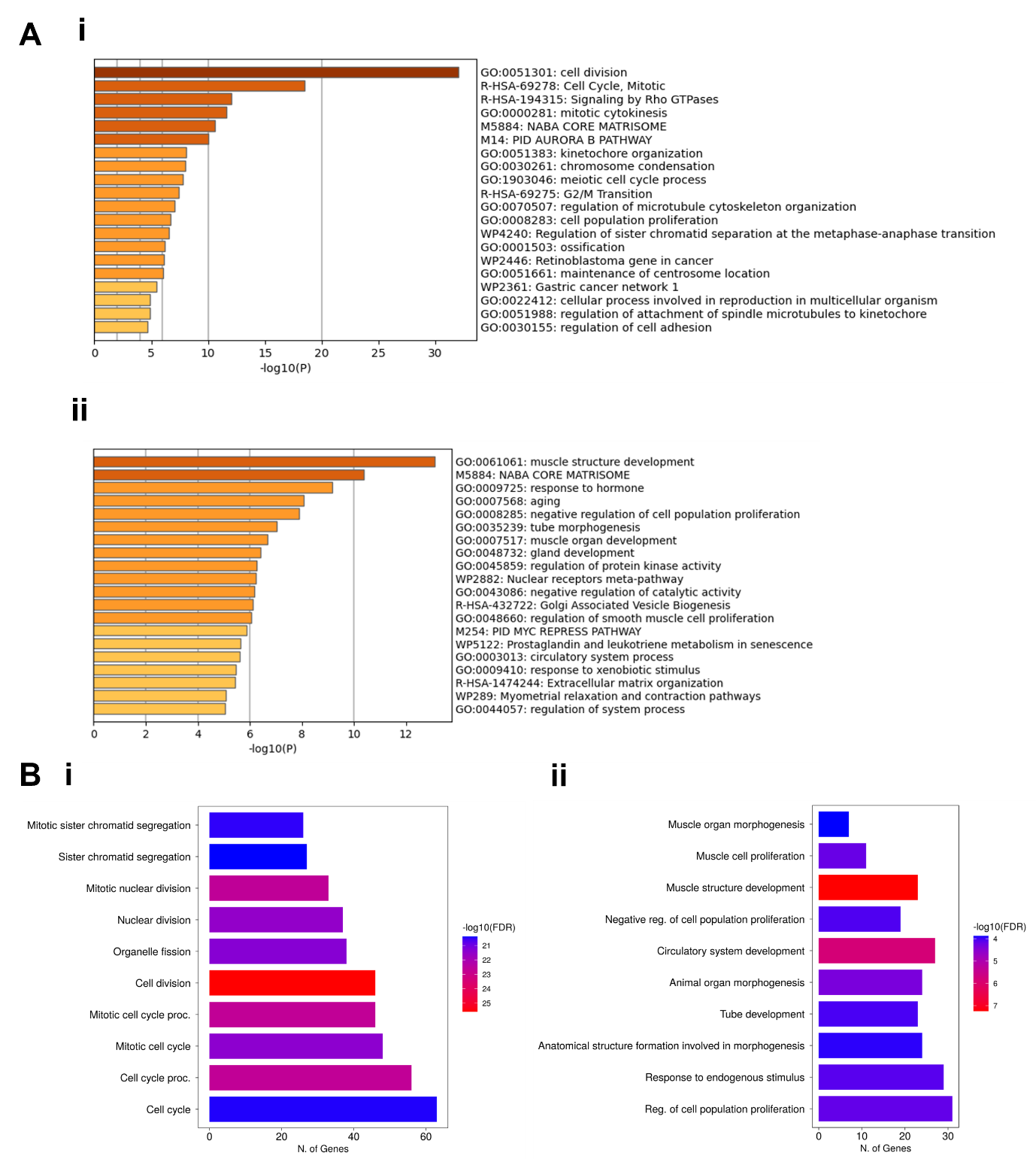
**

**Figure S1.** Enrichment analysis by A) Metascape and B) Gene Ontology of i) upregulated and ii) downregulated genes in MSCs expanded on a tropoelastin substrate, compared to control MSCs grown on tissue culture plastic.

**
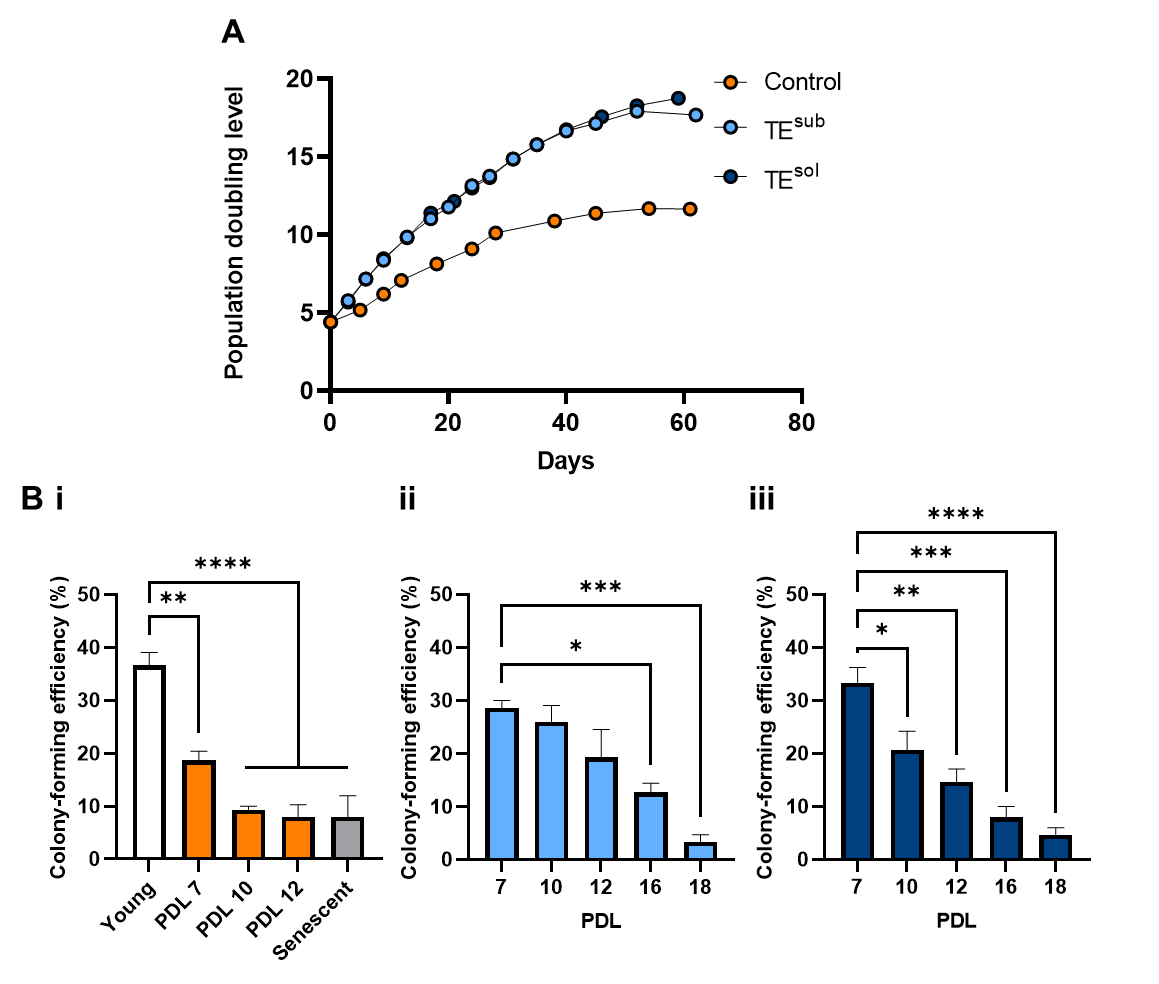
Figure S2.** A) Population doubling kinetics of MSCs cultured with and without tropoelastin supplementation either as a substrate coating (TE^sub^) or as a soluble additive (TE^sol^). B) Colony-forming potential of i) control, ii) TE^sub^, and iii) TE^sol^ MSCs at different population doubling levels (PDLs). Clonogenic assay was performed with three replicates. * P< 0.05; ** P<0.01; *** P<0.001; **** P<0.0001.

**
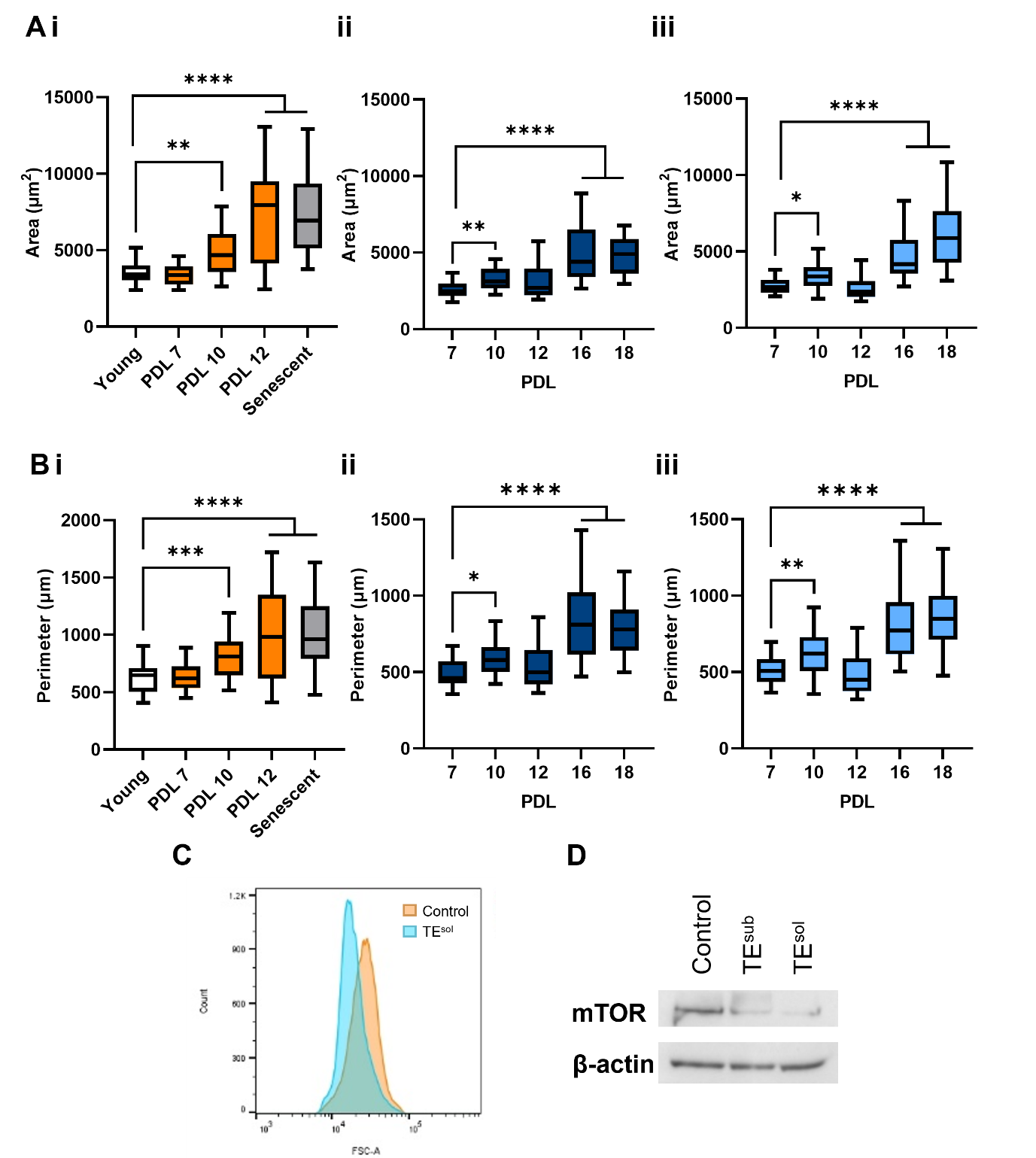
**

**Figure S3.** Cell morphology of MSCs. The A) area and B) perimeter of i) control, ii) TE^sub^, and iii) TE^sol^ MSCs increase with increasing population doubling level (PDL). Measurements were performed on fifty cells. C) Forward scatter of PDL 10 MSCs analysed by flow cytometry. D) mTOR protein abundance of control, TE^sub^, and TE^sol^ MSCs. Beta-actin was included as a loading control. * P< 0.05; ** P<0.01; *** P<0.001; **** P<0.0001.

**
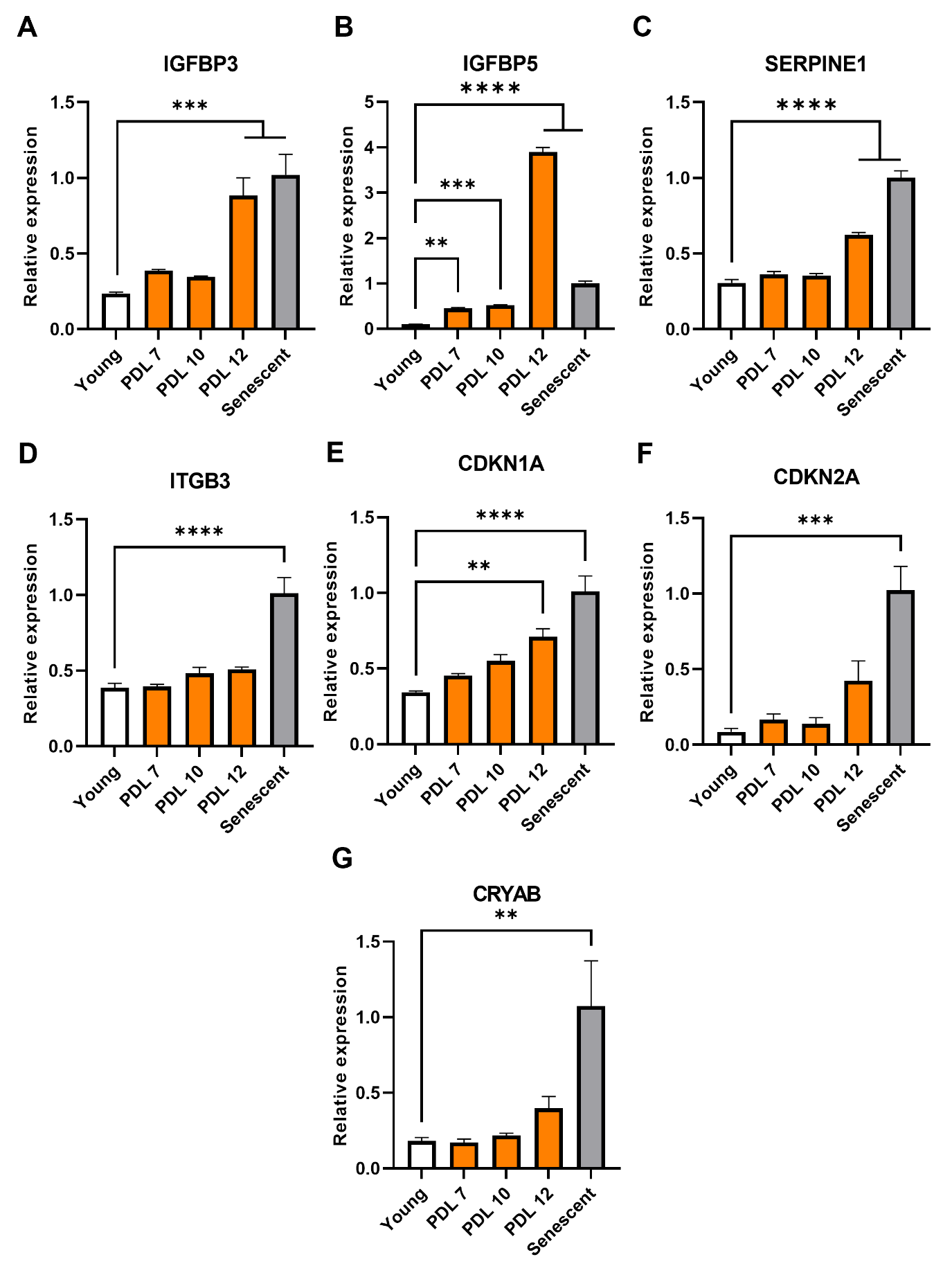
**

**Figure S4.** Expression of senescence-associated molecular markers during MSC aging. Transcript levels of A) IGFBP3, B) IGFBP5, C) SERPINE1, D) ITGB3, E) CDKN1A, F) CDKN2A, and G) CRYAB increase with increasing population doubling level (PDL). Experiments were performed with three replicates. ** P<0.01; *** P<0.001; **** P<0.0001.

**
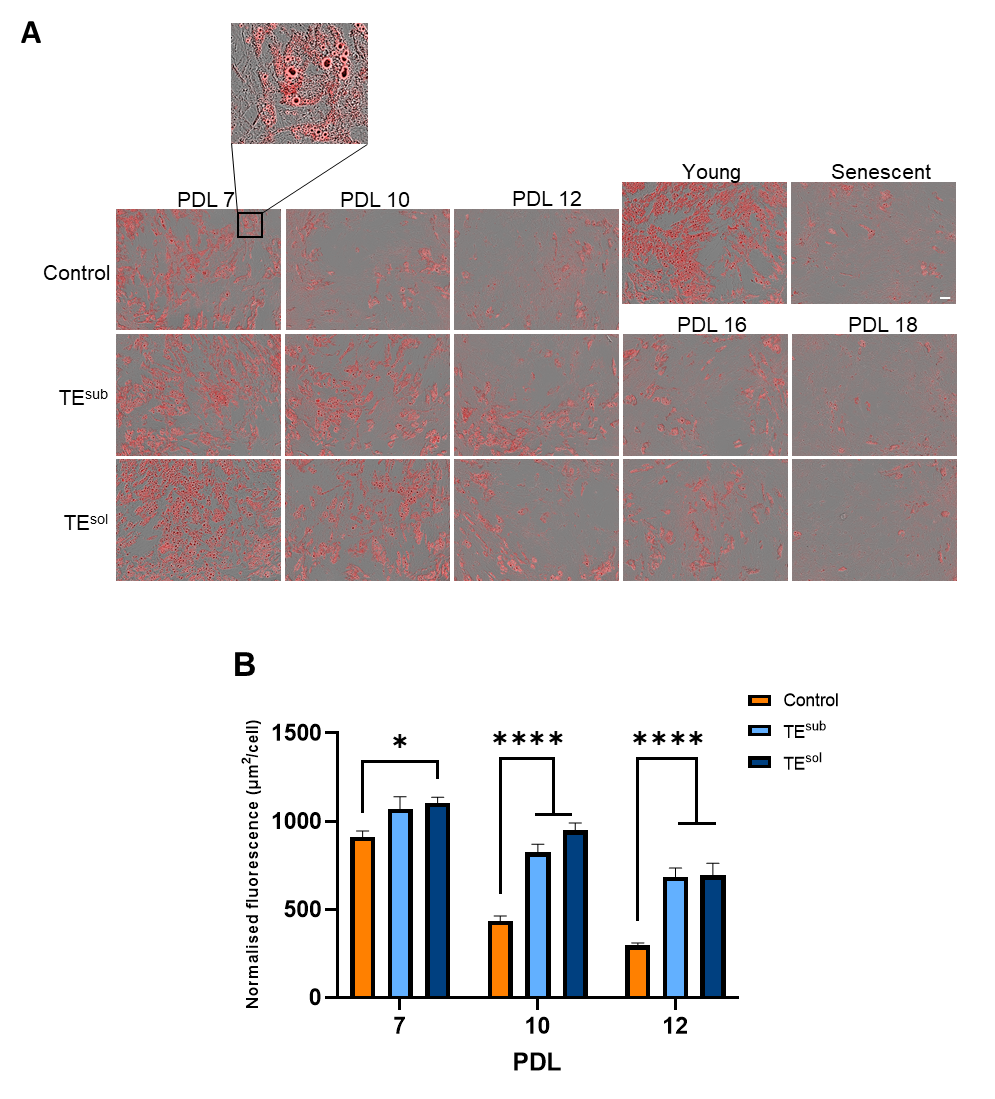
**

**Figure S5.** Adipogenic differentiation potential of MSCs at different replicative ages. A) Representative images of PDL-matched control and tropoelastin-expanded MSCs after adipogenic differentiation. Intracellular lipid droplets were stained with Oil red O. Images were generated by overlaying the orange fluorescence from lipid staining on phase contrast images of the cells. Scale bar = 100 μm. B) Adipogenic differentiation potential of TE^sub^ and TE^sub^ MSCs compared to age-matched controls, quantified via absorbance readings of the solubilised Oil Red O stain. Experiments were conducted with three replicates. * P< 0.05; **** P<0.0001.

**
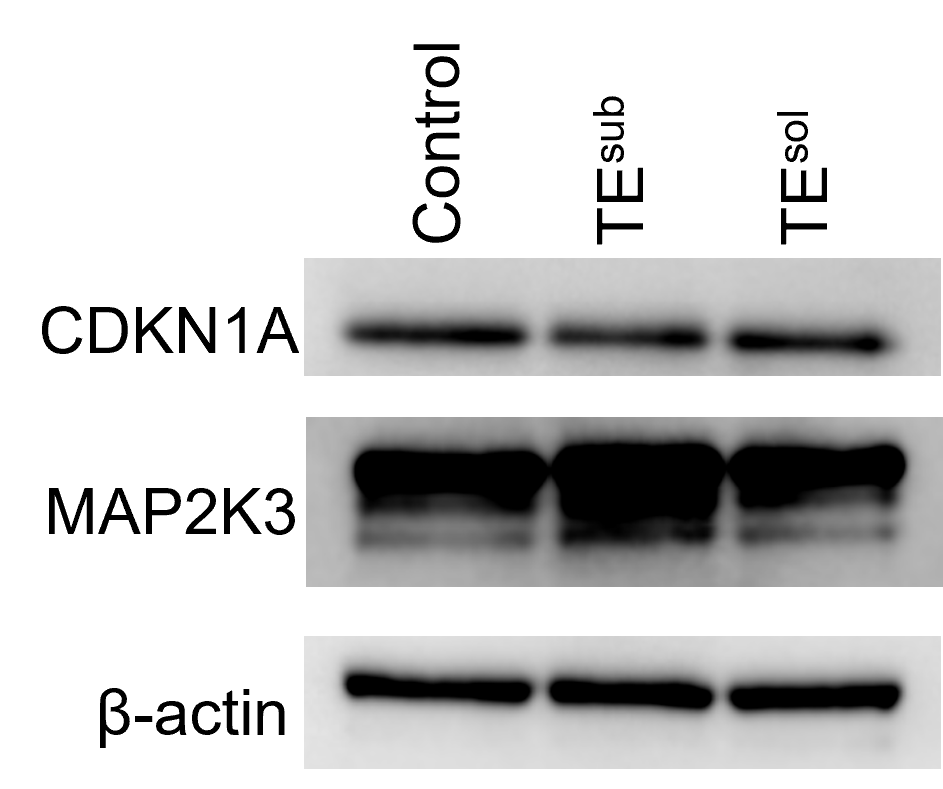
**

**Figure S6. Senescence protein expression in MSCs after short-term tropoelastin exposure.** CDKN1A and MAP2K3 levels of control MSCs, compared to MSCs cultured on tropoelastin substrate (TE^sub^), or in soluble tropoelastin (TE^sol^) for 3 days. Beta-actin was included as a loading control.

**
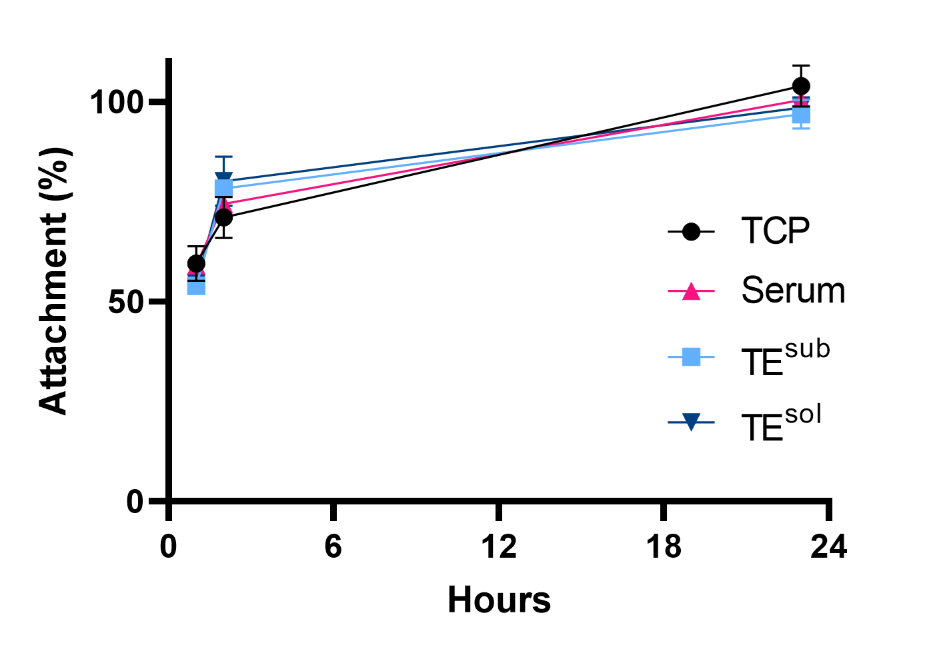
**

**Figure S7.** Adhesion efficiency of MSCs. Attachment of MSCs on tissue culture plastic (TCP), serum-coated TCP, tropoelastin-coated TCP (TE^sub^) or in tropoelastin-supplemented medium (TE^sol^) up to 23 hours post-seeding.

**Table S1.** Genes differentially expressed by TE^sub^ identified in bulk RNA-sequencing.

| Genes | Fold change | padj |
| --- | --- | --- |
| TM4SF20 | 0.407197 | 0.000514 |
| ACTC1 | 0.520682 | 0.003784 |
| PTGDS | 0.563612 | 0.004088 |
| COMP | 0.616355 | 5.08E-14 |
| CRLF1 | 0.620208 | 3.10E-08 |
| ACAN | 0.655825 | 1.73E-31 |
| KRT34 | 0.681367 | 6.90E-12 |
| SYNPO2 | 0.696642 | 4.72E-12 |
| TMEM178B | 0.704411 | 0.008742 |
| HSPB7 | 0.706142 | 2.04E-08 |
| KCND3 | 0.707558 | 0.00067 |
| PAPPA | 0.726913 | 5.72E-13 |
| FNDC1 | 0.730108 | 3.43E-27 |
| OXTR | 0.740071 | 0.000182 |
| CRYAB | 0.747112 | 2.93E-14 |
| DYSF | 0.753376 | 1.08E-05 |
| PTGES | 0.756713 | 1.79E-06 |
| HAPLN3 | 0.75852 | 0.002532 |
| LINC00968 | 0.75976 | 0.000187 |
| COL11A1 | 0.773084 | 1.40E-07 |
| CYP1B1 | 0.776846 | 3.51E-14 |
| HSPB8 | 0.778119 | 0.004933 |
| ID2 | 0.778353 | 1.20E-05 |
| ELN | 0.778527 | 5.98E-10 |
| GPRC5A | 0.783931 | 0.000166 |
| TGFBR1 | 0.784331 | 8.23E-10 |
| PID1 | 0.787078 | 0.001851 |
| FSTL3 | 0.79605 | 1.44E-06 |
| ADAMTS5 | 0.796355 | 2.06E-05 |
| KRT81 | 0.798032 | 0.002535 |
| C6orf132 | 0.798197 | 0.003389 |
| TPD52L1 | 0.799756 | 0.005588 |
| FTL | 0.802902 | 1.10E-12 |
| UCHL1 | 0.803365 | 4.46E-06 |
| CNN1 | 0.803959 | 4.99E-07 |
| MARCHF4 | 0.805043 | 4.28E-06 |
| RCAN1 | 0.816301 | 2.35E-10 |
| FLNC | 0.816419 | 4.29E-05 |
| RHOB | 0.823315 | 3.11E-05 |
| THBS3 | 0.826497 | 0.000465 |
| SLC30A1 | 0.82766 | 0.000302 |
| MCAM | 0.828526 | 8.84E-08 |
| ARL4C | 0.828552 | 0.000304 |
| INKA2 | 0.829024 | 0.000574 |
| MAP2K3 | 0.830769 | 2.02E-05 |
| MFAP5 | 0.832466 | 5.74E-07 |
| SIRPA | 0.835117 | 2.37E-07 |
| IGFBP2 | 0.837453 | 0.000705 |
| ADCY9 | 0.84089 | 0.000701 |
| TXNRD1 | 0.842541 | 6.36E-07 |
| VSIR | 0.842894 | 0.001044 |
| EPAS1 | 0.842954 | 1.41E-06 |
| CITED2 | 0.844668 | 0.00067 |
| ADARB1 | 0.844773 | 0.000444 |
| TRIB3 | 0.845737 | 0.001185 |
| TMEM47 | 0.845759 | 1.69E-05 |
| DHCR24 | 0.846726 | 2.88E-05 |
| ATP10D | 0.851942 | 0.002304 |
| SORT1 | 0.853677 | 4.19E-05 |
| LMCD1 | 0.855597 | 0.004206 |
| TGFB2 | 0.855803 | 0.002725 |
| ZFAS1 | 0.856843 | 0.008833 |
| LIMCH1 | 0.857046 | 0.002532 |
| CALHM5 | 0.8577 | 0.004262 |
| KRT7 | 0.857703 | 0.005059 |
| DACT1 | 0.858071 | 4.99E-05 |
| THBS1 | 0.858606 | 4.41E-05 |
| C1orf198 | 0.859758 | 6.50E-05 |
| FTH1 | 0.859802 | 0.000153 |
| NIPAL3 | 0.862921 | 0.002351 |
| DKK1 | 0.864563 | 0.001211 |
| ATP2B4 | 0.864571 | 0.000501 |
| PDE1C | 0.867149 | 0.001686 |
| CDKN1A | 0.868298 | 1.36E-05 |
| SPCS3 | 0.871202 | 0.002254 |
| ATP10A | 0.872776 | 0.005268 |
| IGF2R | 0.872927 | 0.007852 |
| KCTD20 | 0.873073 | 0.00012 |
| STAT1 | 0.87386 | 0.000182 |
| SLC12A4 | 0.874046 | 0.002747 |
| MGLL | 0.878827 | 0.009791 |
| NQO1 | 0.880209 | 4.44E-05 |
| MFGE8 | 0.880538 | 0.000642 |
| SEL1L3 | 0.882259 | 0.007258 |
| KCTD12 | 0.885394 | 0.006549 |
| PGD | 0.885992 | 0.005435 |
| IGFBP5 | 0.886153 | 0.005285 |
| TALDO1 | 0.891979 | 0.00652 |
| TGM2 | 0.894263 | 0.004622 |
| DDAH1 | 0.89861 | 0.005322 |
| CCND1 | 0.90269 | 0.00579 |
| IGFBP3 | 1.107717 | 0.004917 |
| PGK1 | 1.111383 | 0.003389 |
| TGFBI | 1.117739 | 0.000343 |
| COL8A1 | 1.124561 | 0.003062 |
| EWSR1 | 1.133742 | 0.007258 |
| RBMX | 1.133814 | 0.002304 |
| SCARA3 | 1.134535 | 0.004065 |
| ANPEP | 1.142378 | 0.0002 |
| LGR4 | 1.142986 | 0.008573 |
| NSD2 | 1.144195 | 0.00079 |
| CDC20 | 1.14597 | 0.004643 |
| CKAP5 | 1.147487 | 0.005352 |
| ANLN | 1.148272 | 0.000304 |
| TGFBR2 | 1.149838 | 0.000984 |
| NCAPD2 | 1.150558 | 0.000701 |
| TACC3 | 1.151422 | 0.00652 |
| TPX2 | 1.153947 | 0.000304 |
| TNC | 1.154477 | 0.000228 |
| RACGAP1 | 1.158987 | 0.002418 |
| RBM25 | 1.162107 | 0.009589 |
| NUSAP1 | 1.165014 | 0.008015 |
| CCNB2 | 1.168157 | 0.009246 |
| CKAP2 | 1.171254 | 0.000232 |
| TOP2A | 1.174094 | 6.67E-05 |
| KIF23 | 1.176537 | 0.000376 |
| CTHRC1 | 1.176749 | 0.002527 |
| CTSL | 1.177668 | 0.000741 |
| ECM1 | 1.177787 | 0.006203 |
| LMNB1 | 1.184614 | 0.000289 |
| NUCB2 | 1.184693 | 0.004296 |
| CEP55 | 1.186789 | 0.000994 |
| CCN4 | 1.186913 | 7.46E-05 |
| BHLHE40 | 1.190186 | 0.000175 |
| COL6A3 | 1.191443 | 5.35E-05 |
| LAMB1 | 1.19226 | 6.07E-06 |
| KTN1 | 1.192546 | 0.00264 |
| ACIN1 | 1.193221 | 0.000994 |
| GPSM2 | 1.193564 | 0.005994 |
| CLDN11 | 1.194207 | 5.35E-05 |
| NCAPG2 | 1.197202 | 0.000643 |
| LUC7L3 | 1.197542 | 0.008892 |
| SPAG5 | 1.198316 | 0.000232 |
| PIM1 | 1.19937 | 0.008049 |
| BUB1B | 1.200474 | 0.001873 |
| KIF4A | 1.200831 | 0.001403 |
| UACA | 1.20278 | 0.006624 |
| CKAP2L | 1.207112 | 0.001403 |
| SLIT3 | 1.210023 | 9.94E-08 |
| BUB1 | 1.210663 | 0.000181 |
| GOLGB1 | 1.210678 | 0.002669 |
| RBBP6 | 1.210833 | 0.007068 |
| GDF5 | 1.211773 | 0.002669 |
| GCC2 | 1.215101 | 0.006708 |
| ODF2 | 1.219698 | 0.000934 |
| CXCL12 | 1.221085 | 1.41E-09 |
| PRC1 | 1.221743 | 2.82E-07 |
| KNL1 | 1.222802 | 0.000343 |
| TPR | 1.223907 | 0.000304 |
| CCNB1 | 1.224095 | 1.99E-08 |
| CCDC88A | 1.229863 | 0.000169 |
| CIT | 1.231534 | 7.78E-05 |
| AKAP9 | 1.236956 | 0.004917 |
| BRD8 | 1.23736 | 0.000732 |
| S100A10 | 1.237519 | 7.42E-10 |
| MPHOSPH10 | 1.238827 | 0.009684 |
| NDC80 | 1.24833 | 0.002038 |
| SMC4 | 1.253246 | 7.80E-07 |
| HMGB2 | 1.257198 | 9.35E-08 |
| NKTR | 1.257275 | 0.003071 |
| GOLGA4 | 1.261729 | 0.000489 |
| LRRC15 | 1.263297 | 0.000254 |
| HMMR | 1.269148 | 0.00039 |
| MALAT1 | 1.274149 | 0.005891 |
| PRR11 | 1.276473 | 2.04E-08 |
| PLAU | 1.281345 | 3.83E-05 |
| WSB1 | 1.286823 | 0.000348 |
| TMEM119 | 1.288846 | 2.52E-11 |
| CRYBG1 | 1.289377 | 0.000701 |
| KIF14 | 1.289539 | 0.001166 |
| ENPP2 | 1.293079 | 5.90E-06 |
| CD82 | 1.300604 | 0.00151 |
| ASPM | 1.303461 | 5.16E-09 |
| OLFML2B | 1.307494 | 4.29E-05 |
| KIF20A | 1.307562 | 1.07E-12 |
| BOD1L1 | 1.309419 | 0.0002 |
| LAMA4 | 1.313246 | 3.47E-09 |
| KIF20B | 1.315476 | 9.32E-06 |
| DLGAP5 | 1.315852 | 1.55E-08 |
| ANKRD12 | 1.337444 | 0.002046 |
| TNFRSF19 | 1.339355 | 0.001193 |
| CCDC34 | 1.357849 | 0.002127 |
| MKI67 | 1.361931 | 1.26E-14 |
| SGO2 | 1.363338 | 9.94E-08 |
| MATN2 | 1.371376 | 0.000175 |
| STC1 | 1.374429 | 0.006734 |
| CENPF | 1.39275 | 1.24E-10 |
| CEP290 | 1.4113 | 0.004529 |
| MEG3 | 1.424566 | 5.38E-07 |
| ABI3BP | 1.442839 | 0.00776 |
| NEAT1 | 1.448872 | 1.06E-06 |
| NFKBIZ | 1.482461 | 0.005268 |
| DNM3OS | 1.500681 | 0.000304 |
| CD74 | 1.518499 | 0.002402 |
| TRNH | 1.567339 | 0.005285 |
| CENPE | 1.601154 | 5.51E-12 |
| AHSA2P | 1.686723 | 0.008022 |
| COLEC12 | 1.758137 | 3.43E-27 |
| GOLGA8A | 1.863496 | 0.007522 |
| CH25H | 1.997201 | 4.44E-05 |

**Table S2.** Youth-associated proteins upregulated in young versus senescent MSCs, and their function, localisation, and link to senescence.

| Gene | Function | Canonical localisation | Link to senescence |
| --- | --- | --- | --- |
| BGN | May be involved in collagen fibre assembly | Extracellular space | SASP factor;[1-3] pro-inflammatory[4] |
| CFL1 | Regulates actin filament dynamics, cell morphology, and cytoskeletal organisation | Cytoplasmic | Increased with aging[5] |
| COL12A1 | Matrix component | Extracellular space | SASP factor[2, 3] |
| COL1A2 | Matrix component | Extracellular space | SASP factor[3] |
| EEF1A1.EEF1A1P5 | Protein synthesis | Cytoplasmic/ nuclear | Overexpression of Drosophila homolog EF1a extends lifespan[6] |
| ENO1 | Glucose metabolism; plasminogen activator | Cytoplasmic; Extracellular space | SASP factor[2] |
| GAPDH | Glucose metabolism | Cytoplasmic | SASP factor;[2]  depletion causes senescence in cancer cell line[7] |
| IGFBP6 | Insulin-like growth factor modulation | Extracellular space | SASP factor;[8]  overexpression increases replicative lifespan of fibroblasts[9] |
| LMNA | Nuclear envelope protein | Cytoplasmic; nuclear | Accumulates during aging[10] |
| MIF | Cytokine involved in innate immune response | Extracellular space | SASP factor[2]  Suppresses senescence in fibroblasts[11] |
| MMP2 | Metalloproteinase | Extracellular space | SASP factor;[2, 8, 12]  decrease in the brain is associated with neurodegeneration[13] |
| MOS | Cell cycle protein | Cytoplasmic |  |
| PCOLCE | Enhances procollagen C-proteinase activity | Extracellular space |  |
| PENK | Neurotransmitter | Extracellular space | Anti-inflammatory secreted protein[14] |
| PPIA | Isomerization of proline imidic peptide bonds in oligopeptides | Cytoplasmic; Extracellular space | SASP factor;[2, 3] involved in inflammation signalling[15] |
| TMSB10 | Cytoskeleton organisation | Cytoskeletal |  |

**Table S3.** SASP proteins upregulated in senescent versus young MSCs, and their function, localisation, and link to senescence.

| Gene | Function | Canonical localisation | Link to senescence |
| --- | --- | --- | --- |
| BMP1 | Metalloprotease | Extracellular space |  |
| CALU | Calcium binding | Cytoplasmic | SASP factor[2] |
| CST3 | Cysteine protease inhibitor | Extracellular space | SASP factor[1, 2, 8] |
| CTSB | Thiol protease; Collagen catabolism | Extracellular space | SASP factor[2, 8] |
| EFEMP1 | EGF signalling | Extracellular space | Overexpression causes senescence in hepatocellular carcinoma cells[16] |
| FSTL1 | Angiogenesis; regulation of the immune response, cell proliferation and differentiation | Extracellular space | Causes senescence in alveolar epithelial cells[17] |
| IGFBP2 | Insulin-like growth factor modulation | Extracellular space | SASP factor[1, 8] |
| IGFBP3 | Insulin-like growth factor modulation | Extracellular space | SASP factor[8, 12, 18] |
| IGFBP7 | Insulin-like growth factor modulation | Extracellular space | SASP factor[1, 3, 8] |
| LOXL2 | Cross-linking of extracellular matrix proteins | Extracellular space | SASP factor[2] |
| PTX3 | Regulation of innate resistance to pathogens and inflammatory reactions | Extracellular space | SASP factor[2] |
| QSOX1 | Facilitates disulfide bond formation in a variety of extracellular proteins | Extracellular space | SASP factor[2] |
| SERPINE1 | Serine protease inhibitor | Extracellular space | SASP factor[8] |
| SPOCK1 | Involved in cell-cell and cell-matrix interactions. | Extracellular space |  |
| THBS1 | Mediates cell-to-cell and cell-to-matrix interactions; involved in inflammation | Extracellular space | SASP factor[18] |
| TIMP1 | Metalloproteinase inhibitor | Extracellular space | SASP factor[1, 2, 12] |
| VIM | Filaments found in various non-epithelial cells, especially mesenchymal cells | Cytoplasmic | SASP factor[2, 3] |

**Table S4.** Secreted proteins differentially produced by TE^sub^ MSCs compared to age-matched control cells, and their function, localisation, and link to senescence.

| Gene | Function | Canonical localisation | Link to senescence |
| --- | --- | --- | --- |
| COL5A1 | Matrix component | Extracellular space |  |
| ECM1 | Negative regulator of bone mineralization; inhibition of metalloproteinase | Extracellular space | SASP factor[1, 2] |
| FLNA | Actin binding, involved in cell-cell contacts | Cytoplasmic | SASP factor[3] |
| FBN1 | Part of the microfibrils; modulates availability of TGF-beta | Extracellular space | SASP factor[3] |
| LUM | Regulates collagen fibril assembly | Extracellular space | Uniquely found in young cells compared to replicative aged MSCs[3] |
| TIMP2 | Inactivates metalloproteinase | Extracellular space | SASP factor[1, 2, 8, 12] |

**Table S5.** Secreted proteins differentially produced by TE^sol^ MSCs compared to age-matched control cells, and their function, localisation, and link to senescence.

| Gene | Function | Canonical localisation | Relation to senescence |
| --- | --- | --- | --- |
| ACTN4.ACTN1 | Actin binding; transport of structures | Cytoplasmic | SASP factor[2, 12] |
| FLNA | Actin binding; involved in cell-cell contacts | Cytoplasmic | SASP factor[3] |
| SH3BGRL3 | Modulator of glutaredoxin biological activity | Cytoplasmic | SASP factor[2] |
| TPM4 | Binds calcium; binds to actin filaments | Cytoskeleton |  |
| COL3A1 | Matrix component | Extracellular space | SASP factor[12] |

**Supplementary Methods**

*Flow cytometry*

PDL 10 TE^sol^ MSCs were harvested by trypsinisation and resuspended in 5% (v/v) MSC-FBS in PBS at 100,000 cells/ 250 μL and filtered through a 35 μm mesh. Samples were stained with 600 nM propidium iodide (BD Bioscience), 50,000 cells were measured by a BD LSR II Flow Cytometer. Singlets and live cells by propidium iodide staining were gated prior to forward scattering analysis in FlowJo software.

*Western blot*

MSCs were seeded at 5,000 cells cm^−2^ in standard media on TCP, in standard media on TCP coated with 20 μg mL^−1^ tropoelastin in PBS at 4°C overnight (TE^sub^), or in standard media supplemented with 20 μg mL^−1^ recombinant wild-type human tropoelastin on TCP blocked with standard media at 4°C overnight (TE^sol^). After three days, cells were washed with PBS and lysed with RIPA buffer (Thermo Fisher) supplemented with Pierce protease and phosphatase inhibitor (Thermo Fisher). Cell debris was pelleted at 12,000 x *g* for 15 min, and protein concentration was estimated via a Pierce bicinchoninic acid assay (Thermo Fisher). Total protein (5 μg) was denatured at 85°C for 10 min, separated by 4-12% Bis-Tris SDS-PAGE (Thermo Fisher), then transferred to a polyvinylidene fluoride membrane (Millipore). The membrane was blocked with 5% (w/v) skim milk in Tris-buffered saline with Tween 20 (TBST) (25 mM Tris-HCl, pH 7.6, 140 mM NaCl, 0.1% Tween 20) for 1 hr at room temperature and incubated with mTOR (1 in 1000, Cell Signalling Technology #2983) or beta-actin (1 in 1000, Cell Signalling technology #4970) antibodies at 4 °C overnight. After three washes with TBST, the membranes were incubated with horseradish peroxidase (HRP)-conjugated anti-rabbit IgG antibody (1 in 5000, Cell Signalling Technology #7074) for 1 hr at room temperature. Blots were washed three times with TBST, developed with Immobilon ECL Ultra Western HRP Substrate (Millipore) and imaged using the ChemiDoc MP Imaging System (Bio-Rad).

For CDKN1A and MAP2K3, cells were similarly treated and prepared. Membranes were incubated with CDKN1A (1 in 1000, Cell Signalling Technology #2947) or MKK3 (1 in 1000, Cell Signalling Technology #8535) antibodies. Protein levels were probed in a similar manner as described with HRP-conjugated anti-rabbit IgG antibody. Membrane was stripped and incubated with beta-actin antibody at 4 °C overnight and probed in a similar manner as described with HRP-conjugated anti-rabbit IgG antibody.

*Adipogenic differentiation*

Cells were plated at 21,000 cells/cm^2^ and cultured for 5 days in standard media. Using adipogenic induction and maintenance media (Lonza), MSCs were differentiated according to the manufacturer’s instructions. At the end of the differentiation period, cells were washed with PBS twice and fixed with 10% (v/v) formalin for 45 min at room temperature. Cells were stained with NucBlue Live ReadyProbes Reagent (Thermo Fisher) for 20 min, and three representative images were taken for nuclei counting. Images were analysed using Fiji.

Cells were washed twice with Milli-Q water and incubated in 60% (v/v) isopropanol for 5 min. Cells were then stained with 1.8 mg/mL Oil Red O in 60% (v/v) isopropanol for 20 min. Excess stain was washed three times with Milli-Q water before imaging with the orange channel of the IncuCyte platform. Fluorescence area of images was analysed by the IncuCyte. Normalised fluorescence was calculated by dividing the total fluorescence area by the average nuclei number in each sample.

*Adhesion assay*

MSCs were seeded at 5,000 cells/cm^2^ on TCP, TCP pre-coated with standard media, TCP pre-coated with 20 μg/mL tropoelastin as substrate in standard media, or in standard media supplemented with 20 μg/mL soluble tropoelastin on TCP pre-coated with standard media. Cells were washed with PBS and fixed with 4% (v/v) formaldehyde at 1, 5, and 23 hours post-seeding. Fixed cells were stained with 0.1% (w/v) crystal violet in 0.2 M 2-(N-morpholino)ethanesulfonic acid (MES) buffer, pH 5.5, for 1 hr. Excess stain was washed off with Milli-Q water three times. Crystal violet dye was solubilised with 10% (v/v) acetic acid and absorbance was measured at 570 nm using the Tecan Infinite M1000 Pro plate reader. Attachment percentage was calculated by normalising to TCP at 23 hours.

**References**

1. Evans, D.S., et al., *Proteomic Analysis of the Senescence Associated Secretory Phenotype (SASP): GDF-15, IGFBP-2, and Cystatin-C Are Associated with Multiple Aging Traits.* The Journals of Gerontology: Series A, 2023.

2. Basisty, N., et al., *A proteomic atlas of senescence-associated secretomes for aging biomarker development.* PLOS Biology, 2020. **18**(1): p. e3000599.

3. Severino, V., et al., *Insulin-like growth factor binding proteins 4 and 7 released by senescent cells promote premature senescence in mesenchymal stem cells.* Cell Death & Disease, 2013. **4**(11): p. e911-e911.

4. Schaefer, L., et al., *The matrix component biglycan is proinflammatory and signals through Toll-like receptors 4 and 2 in macrophages.* The Journal of Clinical Investigation, 2005. **115**(8): p. 2223-2233.

5. Tsai, C.-H., et al., *Up-regulation of cofilin-1 in cell senescence associates with morphological change and p27kip1-mediated growth delay.* Aging Cell, 2021. **20**(1): p. e13288.

6. Shepherd, J.C., et al., *Fruit flies with additional expression of the elongation factor EF-1 alpha live longer.* Proceedings of the National Academy of Sciences, 1989. **86**(19): p. 7520-7521.

7. Phadke, M., et al., *Accelerated cellular senescence phenotype of GAPDH-depleted human lung carcinoma cells.* Biochemical and Biophysical Research Communications, 2011. **411**(2): p. 409-415.

8. Saul, D., et al., *A new gene set identifies senescent cells and predicts senescence-associated pathways across tissues.* Nature Communications, 2022. **13**(1): p. 4827.

9. Micutkova, L., et al., *Insulin-like growth factor binding protein-6 delays replicative senescence of human fibroblasts.* Mechanisms of Ageing and Development, 2011. **132**(10): p. 468-479.

10. Primmer, S.R., et al., *Lamin A to Z in normal aging.* Aging (Albany NY), 2022. **14**(20): p. 8150-8166.

11. Hudson, J.D., et al., *A Proinflammatory Cytokine Inhibits P53 Tumor Suppressor Activity.* Journal of Experimental Medicine, 1999. **190**(10): p. 1375-1382.

12. Ozcan, S., et al., *Unbiased analysis of senescence associated secretory phenotype (SASP) to identify common components following different genotoxic stresses.* Aging (Albany NY), 2016. **8**(7): p. 1316-29.

13. Brkic, M., et al., *Friends or Foes: Matrix Metalloproteinases and Their Multifaceted Roles in Neurodegenerative Diseases.* Mediators of Inflammation, 2015. **2015**: p. 620581.

14. Milwid, J.M., et al., *Enriched Protein Screening of Human Bone Marrow Mesenchymal Stromal Cell Secretions Reveals MFAP5 and PENK as Novel IL-10 Modulators.* Molecular Therapy, 2014. **22**(5): p. 999-1007.

15. Nigro, P., et al., *Cyclophilin A is an inflammatory mediator that promotes atherosclerosis in apolipoprotein E–deficient mice.* Journal of Experimental Medicine, 2010. **208**(1): p. 53-66.

16. Hu, J., et al., *Epidermal growth factor-containing fibulin-like extracellular matrix protein 1 (EFEMP1) suppressed the growth of hepatocellular carcinoma cells by promoting Semaphorin 3B(SEMA3B).* Cancer Medicine, 2019. **8**(6): p. 3152-3166.

17. Sun, W., et al., *FSTL1 promotes alveolar epithelial cell aging and worsens pulmonary fibrosis by affecting SENP1-mediated DeSUMOylation.* Cell Biology International, 2023. **47**(10): p. 1716-1727.

18. Medeiros Tavares Marques, J.C., et al., *Identification of new genes associated to senescent and tumorigenic phenotypes in mesenchymal stem cells.* Scientific Reports, 2017. **7**(1): p. 17837.
